# Soil Responses to Mineral Nitrogen and Carbon-Bearing Amendments in Moso Bamboo Forests: A Meta-Analysis

**DOI:** 10.3390/plants15213301

**Published:** 2026-10-26

**Authors:** Amina Qamar, Nasir Shad, Abbas Ali, Nizar Nawaz, Chunce Guo

**Affiliations:** 1 Jiangxi Provincial Key Laboratory of Improved Variety Breeding and Efficient Utilization of Native Tree Species, College of Forestry, Jiangxi Agricultural University, Nanchang 330045, China; aminaqamar774@gmail.com; 2 School of Ecology and Applied Meteorology, Nanjing University of Information Science & Technology, Nanjing 210044, China; 202351530001@nuist.edu.cn or dearbotanist@gmail.com; 3 Key Laboratory of National Forestry and Grassland Administration on Forest Ecosystem Protection and Restoration of Poyang Lake Watershed, Jiangxi Agricultural University, Nanchang 330045, China; jainkhan619@gmail.com; 4 Jiangxi Provincial Key Laboratory of Subtropical Forest Resources Cultivation, College of Forestry, Jiangxi Agricultural University, Nanchang 330045, China; nizarnawaz66@gmail.com

**Keywords:** *Phyllostachys edulis*, biochar, nitrogen fertilization, soil organic carbon, greenhouse gas emissions, microbial biomass, meta-analysis, dose–response, subtropical forest

## Abstract

Moso bamboo (*Phyllostachys edulis*) plantations in subtropical China are intensively fertilized, yet it remains unclear how soil responses differ between mineral nitrogen and carbon-bearing amendments, and how far they depend on the nitrogen application rate. This meta-analysis of 69 articles covered four fertilization regimes (N, nitrogen alone; NPK, balanced mineral fertilizer; N+OM, nitrogen with organic carbon; B, biochar alone). We found that soil pH and organic carbon (SOC) responded in opposite directions to nitrogen alone and to the carbon-bearing amendments. Nitrogen alone acidified the soil (−5.3%) and reduced SOC (−11.4%), whereas B and N+OM raised pH (+9.5%, +3.4%) and SOC (+14.0%, +28.7%); NPK also raised SOC (+17.9%) but slightly lowered pH (−1.7%). The mineral regimes increased nitrate (+72.9% under N) but not total nitrogen, which rose only under N+OM (+33.3%). Microbial biomass carbon (MBC) increased under N, NPK and N+OM (+9.8% to +20.6%), whereas enzyme activities were largely unchanged. Nitrogen alone increased cumulative nitrous oxide emission (+18.7%) and reduced methane uptake (−15.5%), whereas N+OM lowered the nitrous oxide emission rate (−5.9%) and increased methane uptake (+9.0%). Each additional 100 kg N ha^−1^ yr^−1^ raised MBC by 1.9% and ammonium nitrogen by 4.7% without altering SOC, although most of the application rates tested were below 100 kg N ha^−1^ yr^−1^. Within this range, adding an exogenous carbon source to nitrogen may limit soil acidification and SOC depletion.

## 1. Introduction

Moso bamboo (*Phyllostachys edulis* (Carrière) J. Houzeau) is the most widely planted bamboo in the world [1]. It covers several million hectares in subtropical China, where eddy-covariance measurements indicate that its net ecosystem production exceeds that of most subtropical forest types [2]. Unlike most forests, Moso stands are managed in a manner similar to agricultural cropping systems; deep tillage and repeated fertilization are standard practice in commercial stands [3]. Culm recruitment and shoot yield respond strongly to nutrient supply; growers therefore apply mineral nitrogen annually, often at rates comparable to those used in row-crop agriculture, and sustain the practice for decades [3,4,5]. Repeated nitrogen inputs alter soil carbon fractions and the understory of these stands within a few years [6]. Moso plantations are consequently both a regionally important carbon store and a site of fertilizer-driven biogeochemical change, making the soil consequences of fertilizer application in these plantations a matter of silvicultural and climatic relevance. Such changes are not specific to bamboo: nitrogen addition acidifies soils across terrestrial ecosystems [7], and the effect is pronounced in subtropical forests on acidic, strongly weathered soils of low base saturation [8]. Moso bamboo differs from most fertilized plantations mainly in the intensity of its management, with annual nitrogen application, deep tillage and the removal of shoots and culms on a short cycle [3,9], making its soils a suitable model system for examining fertilizer effects in intensively managed plantations.

The consequences of nitrogen enrichment in forest soils are broadly established. Nitrogen addition acidifies soil [7], alters the readily available inorganic nitrogen pool and accelerates nitrogen loss pathways, with nitrate concentration and leaching among the most responsive variables recorded across terrestrial ecosystems [10]. It alters belowground carbon dynamics by balancing suppressed decomposition and stimulated microbial mining of native organic matter, and soil carbon may accumulate or decline depending on which process prevails [11]. Nitrogen enrichment also weakens the soil methane (CH_4_) sink by shifting the balance between methanotrophs and methanogens, an effect demonstrated directly in a Moso bamboo forest [12]. Microbial responses are not unidirectional: the global average response of microbial biomass to nitrogen addition is a decline [13], yet the response varies strongly with site conditions and is frequently positive where soil pH and the carbon-to-nitrogen (C:N) ratio are moderate [14], even where the activity of the enzymes responsible for organic matter breakdown does not increase [15], suggesting a shift in community allocation rather than uniform stimulation or suppression.

A parallel body of literature addresses the effects of amendments that supply carbon in addition to nutrients. Biochar raises soil pH through its alkalinity, adds directly to the organic carbon stock, and modifies the aeration and moisture regimes that govern trace-gas production [16,17]. Syntheses report substantial reductions in nitrous oxide (N_2_O) emission after biochar addition [18] and variable, frequently positive, effects on the CH_4_ sink [19]. Organic amendments, combined with mineral fertilizer, act through distinct pathways, supplying labile carbon that alleviates the carbon limitation on microbial growth [20]. The two bodies of work describe mechanisms that differ in kind rather than in degree: mineral nitrogen supplied without carbon leaves microbial metabolism carbon-limited, whereas an amendment that carries carbon alters the stoichiometry of the substrate reaching the microbial community [21]. Management scheme and nitrogen inputs interact in Moso plantations to shape all three gas fluxes simultaneously [22]; the three gases should thus not be evaluated independently of the amendment applied.

Published fertilizer experiments in Moso bamboo plantations nevertheless report their treatments by product category as marketed, for example as chemical fertilizer contrasted with biofertilizer [23], as an increasing-rate series of a single branded bamboo-sourced organic fertilizer against an unfertilized control [24], or as a biochar-based sheep-manure organic fertilizer against conventional compound and sheep-manure organic fertilizers [25]; this practice groups amendments by commercial formulation rather than by their action on the soil. Such a framing conflates two properties that act on the soil through different mechanisms: the quantity of mineral nitrogen applied, expressed as the nitrogen application rate in kilograms of nitrogen per hectare per year; and whether the amendment also supplies exogenous carbon. If the mechanisms outlined above apply, mineral nitrogen and carbon-bearing amendments would be expected to change soil acidity and soil carbon in opposite directions, meaning with opposite signs relative to the unfertilized control, whereas the nitrogen application rate would be expected to change mainly the size of the response. Three recent syntheses have addressed parts of this question. One reports that fertilization reduces soil organic carbon (SOC) in bamboo forests overall, and that the reduction is attributable to inorganic nitrogen while organic and compound fertilizers have no detectable effect [26]; a second compares chemical, organic, biochar, biochar-based and silicate fertilizers for their effects on soil carbon dioxide (CO_2_), N_2_O and CH_4_ emissions from bamboo forests [27]; and a global synthesis of nitrogen addition in forests treats bamboo as one forest type and reports that its soils respond differently from those of other forests [28]. These syntheses group treatments by fertilizer product or consider nitrogen addition alone, and each addresses soil carbon, greenhouse gas (GHG) emissions, or both. Three gaps therefore remain. First, to our knowledge no quantitative synthesis has grouped fertilizer treatments applied to Moso bamboo by whether they deliver mineral nitrogen alone or together with an exogenous carbon source. Second, no synthesis has examined the soil, microbial and GHG responses for the same set of fertilizer treatments, leaving the trade-offs among them unassessed. Third, the nitrogen application rate is recorded in most Moso bamboo experiments but has been treated as categories of fertilization level rather than as a continuous moderator; consequently, it remains unclear at which rates responses saturate, plateau or reverse, although this can be estimated by meta-regression [29] and is directly relevant to management.

To address these three gaps, we synthesized 9246 paired observations from 69 articles, with three objectives: to quantify how the soil, microbial and GHG responses differ between fertilization regimes that do and do not carry exogenous carbon; to test the nitrogen application rate as a continuous moderator of those responses; and to examine which responses move together across treatment conditions. We grouped the treatments into four mutually exclusive regimes according to whether they supply mineral nitrogen alone, together with phosphorus and potassium, or together with an exogenous carbon source: mineral nitrogen alone (N), balanced mineral fertilization (NPK), mineral nitrogen combined with an exogenous organic carbon source (N+OM), and biochar alone (B). The N and NPK regimes are referred to throughout as the mineral regimes, and N+OM and B as the carbon-bearing regimes. Comparing N with NPK indicates the effect of balancing mineral nutrition, and comparing either with the carbon-bearing regimes indicates the effect of adding a carbon-bearing amendment, whose carbon is necessarily applied together with its other properties. We tested four hypotheses: (1) Soil acidity and soil carbon respond in opposite directions to mineral nitrogen and to carbon-bearing amendments, and the nitrogen application rate changes the size of these responses more than their direction; balanced mineral nutrition may also raise soil carbon through a plant-mediated route. (2) Mineral regimes promote the readily available nutrient pool, whereas carbon-bearing regimes promote the nutrient stocks. (3) Extracellular enzyme activity tracks the resource that remains limiting after fertilization more closely than microbial biomass does. (4) Greenhouse gas responses are compensatory, such that no regime is favorable for CO_2_, N_2_O, and CH_4_ at the same time. To our knowledge, this is the first synthesis to group fertilizer treatments in Moso bamboo plantations by whether they carry exogenous carbon rather than by commercial fertilizer category, and it is intended to inform the management of the biogeochemical costs of intensive bamboo production.

## 2. Results

### 2.1. Response of Soil Physicochemical Properties and Nutrient Pools to Fertilization Regimes

**Figure 1:**
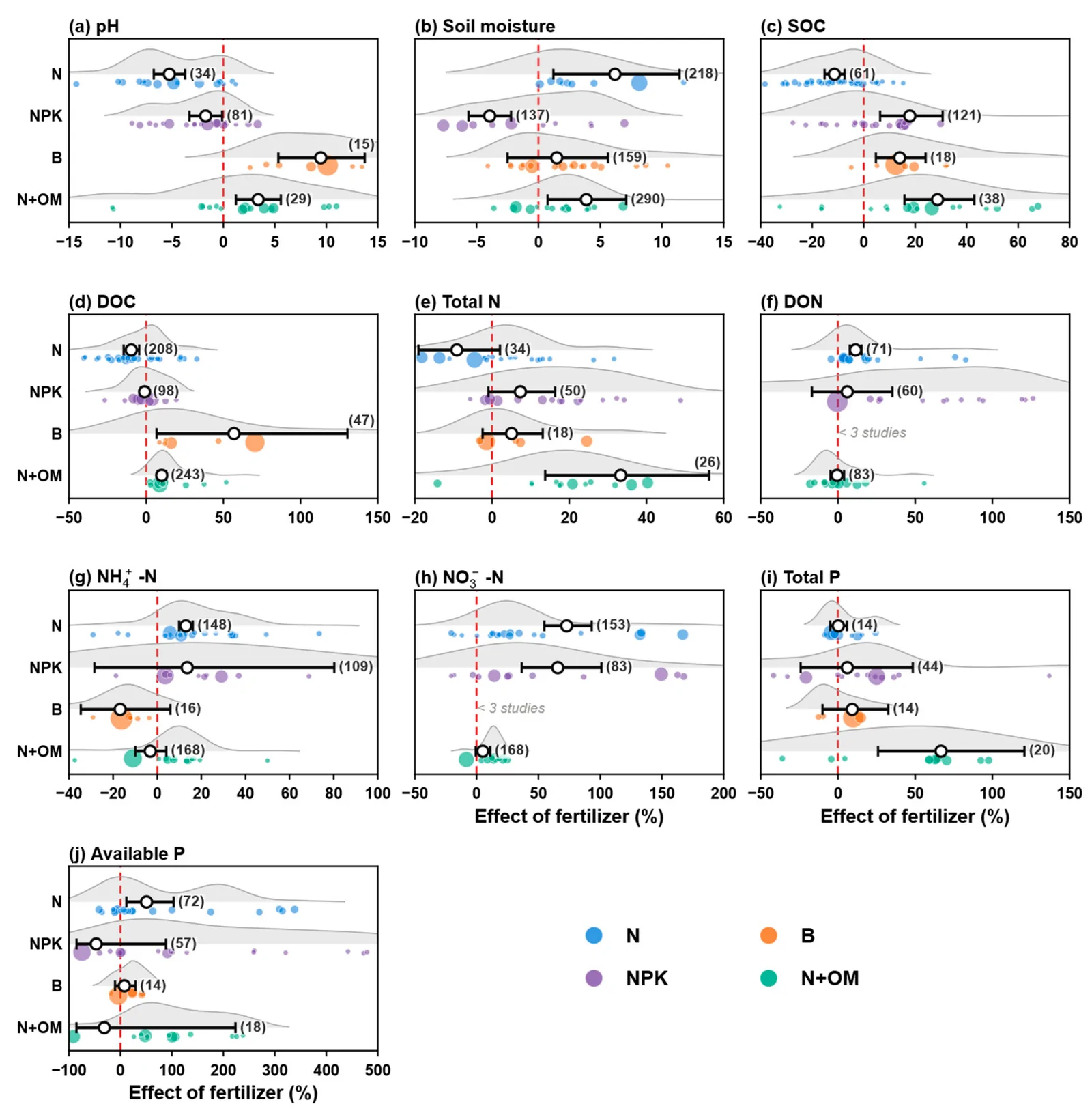
Effect of fertilization regimes on soil physicochemical properties and nutrient pools in Moso bamboo forests. (**a**) pH, (**b**) soil moisture (SM), (**c**) soil organic carbon (SOC), (**d**) dissolved organic carbon (DOC), (**e**) total nitrogen (TN), (**f**) dissolved organic nitrogen (DON), (**g**) ammonium nitrogen (NH_4_^+^-N), (**h**) nitrate nitrogen (NO_3_^−^-N), (**i**) total phosphorus (TP) and (**j**) available phosphorus (AP). Mean effects (open circles) with 95% confidence intervals were considered significant when the interval did not overlap zero (red dashed line). Colored points are the weighted mean effects of the treatment conditions within each study (point size proportional to weight in the meta-analysis), and gray shading shows their distribution. Numbers in parentheses are the number of paired observations retained after outlier screening; “<3 studies” marks combinations not estimated. Abbreviations: N, mineral nitrogen as the only varying input; NPK, balanced mineral fertilization without exogenous carbon; N+OM, mineral nitrogen with an exogenous organic carbon source; B, biochar applied without mineral nitrogen.

Nitrogen alone and the carbon-bearing regimes changed soil acidity and the soil carbon pools in opposite directions (Figure 1). Soil pH decreased under N (−5.3%) and NPK (−1.7%) and increased under B (+9.5%) and N+OM (+3.4%) (Figure 1a). Soil moisture (SM) increased under N (+6.2%) and N+OM (+3.9%) and decreased under NPK (−3.9%) (Figure 1b). Soil organic carbon decreased under N (−11.4%) and increased under NPK (+17.9%), B (+14.0%) and N+OM (+28.7%) (Figure 1c). Dissolved organic carbon (DOC) decreased under N (−9.7%) and increased under B (+56.8%) and N+OM (+10.1%) (Figure 1d). Total nitrogen (TN) increased under N+OM (+33.3%) (Figure 1e), and dissolved organic nitrogen (DON) under N (+11.4%) (Figure 1f). Ammonium nitrogen (NH_4_^+^-N) increased under N (+13.0%) (Figure 1g), and nitrate nitrogen (NO_3_^−^-N), the most responsive soil variable measured, increased under N (+72.9%) and NPK (+65.7%) (Figure 1h). Total phosphorus (TP) increased under N+OM (+66.7%) (Figure 1i), and available phosphorus (AP) under N (+50.8%) (Figure 1j). The remaining effects did not differ from zero, namely SM under B, DOC under NPK, TN under N, NPK and B, DON under NPK and N+OM, NH_4_^+^-N under NPK, B and N+OM, NO_3_^−^-N under N+OM, TP under N, NPK and B, and AP under NPK, B and N+OM (Figure 1).

Among the variables reported in fewer studies, alkali-hydrolyzable nitrogen increased under N+OM (+38.1%) and total potassium under NPK (+5.2%) (Table S1). The regimes differed for pH, SM, SOC, DOC, TN, NO_3_^−^-N and TP (Q_B_, *p* < 0.05; Table S2). For pH, every pair of regimes differed, the decline being stronger under N than under NPK. For SOC, N differed from each of the other three regimes, whereas NPK, B and N+OM did not differ from one another (*p* > 0.05). For NO_3_^−^-N, the two mineral regimes did not differ, and both differed from N+OM (Table S2).

### 2.2. Response of Soil Microbial Biomass, Enzyme Activities and GHG Fluxes to Fertilization Regimes

**Figure 2:**
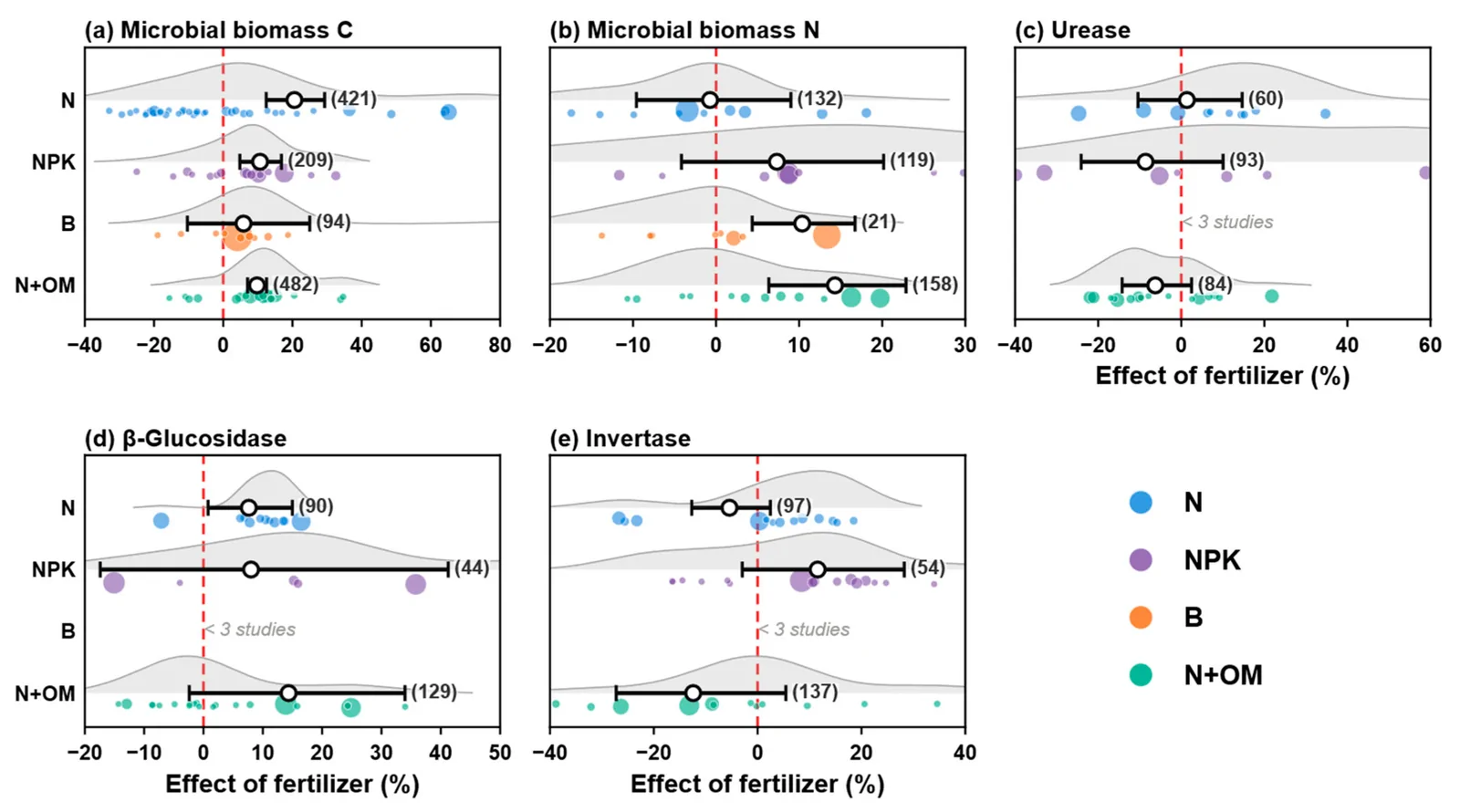
Effect of fertilization regimes on soil microbial biomass and enzyme activities in Moso bamboo forests. (**a**) Microbial biomass carbon (MBC), (**b**) microbial biomass nitrogen (MBN), (**c**) urease, (**d**) β-glucosidase and (**e**) invertase. Mean effects (open circles) with 95% confidence intervals were considered significant when the interval did not overlap zero (red dashed line). Colored points are the weighted mean effects of the treatment conditions within each study (point size proportional to weight in the meta-analysis), and gray shading shows their distribution. Numbers in parentheses are the number of paired observations retained after outlier screening; “<3 studies” marks combinations not estimated. N, mineral nitrogen as the only varying input; NPK, balanced mineral fertilization without exogenous carbon; N+OM, mineral nitrogen with an exogenous organic carbon source; B, biochar applied without mineral nitrogen.

Microbial biomass increased under most regimes, whereas enzyme activities were largely unchanged (Figure 2). Microbial biomass carbon (MBC) increased under N (+20.6%), NPK (+10.6%) and N+OM (+9.8%) (Figure 2a), and microbial biomass nitrogen (MBN) under B (+10.4%) and N+OM (+14.3%) (Figure 2b). Of the hydrolytic enzymes, only β-glucosidase responded, increasing under N (+7.6%) (Figure 2d). Microbial biomass carbon under B, MBN under N and NPK, β-glucosidase under NPK and N+OM, and urease and invertase under every regime did not differ from zero (Figure 2a–e). Phosphatase increased under N (+22.0%) (Table S1).

**Figure 3:**
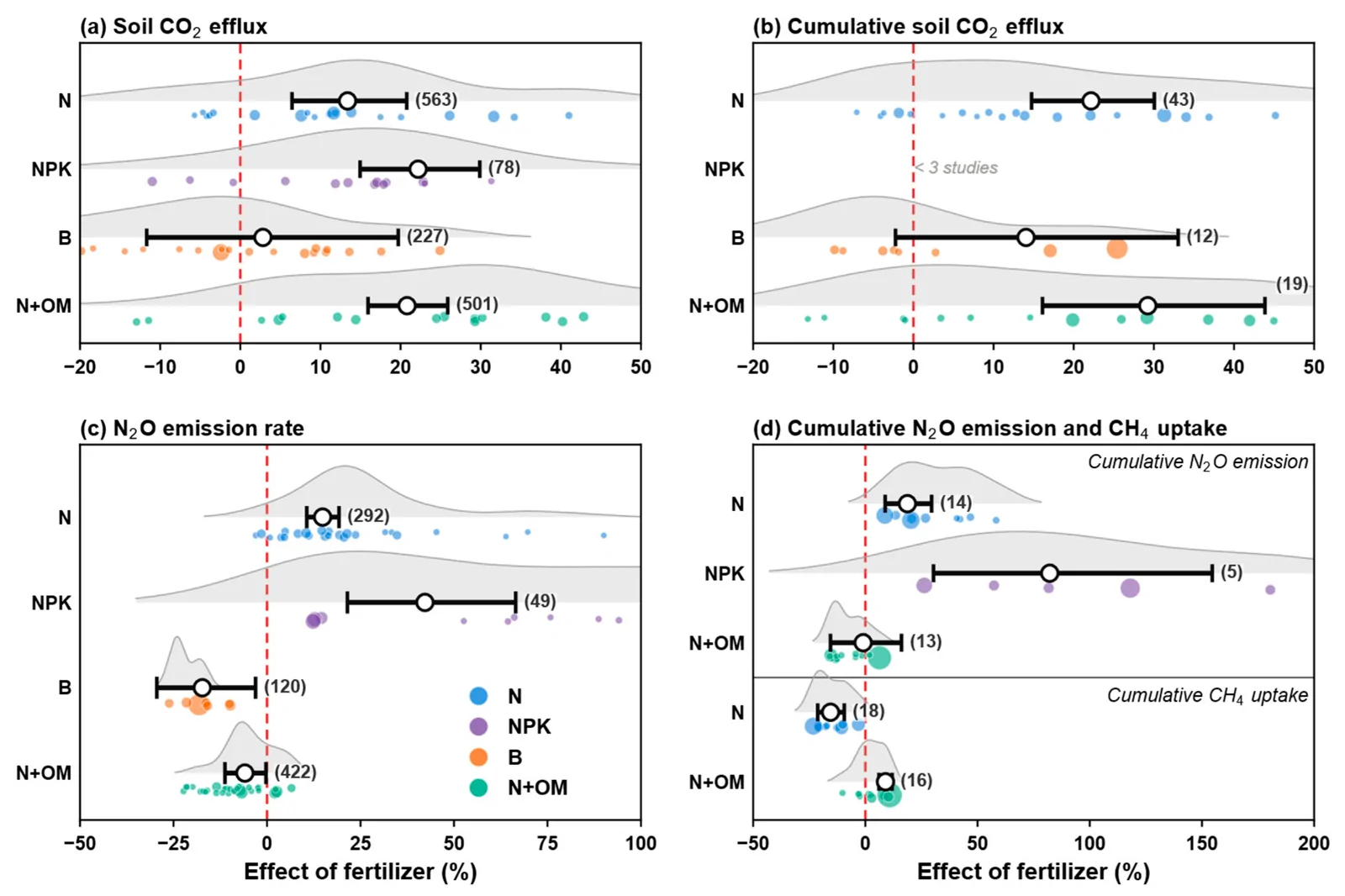
Effect of fertilization regimes on greenhouse gas (GHG) fluxes in Moso bamboo forests. (**a**) Soil CO_2_ efflux, (**b**) cumulative soil CO_2_ efflux, (**c**) N_2_O emission rate, (**d**) cumulative N_2_O emission (upper part) and cumulative CH_4_ uptake (lower part; uptake expressed as a positive quantity), separated by the horizontal black line. Soil CO_2_ efflux combines soil respiration and CO_2_ emission rate, which were measured as total soil-surface efflux in all field studies. Mean effects (open circles) with 95% confidence intervals were considered significant when the interval did not overlap zero (red dashed line). Colored points are the weighted mean effects of the treatment conditions within each study (point size proportional to weight in the meta-analysis), and gray shading shows their distribution. Numbers in parentheses are the number of paired observations retained after outlier screening; “<3 studies” marks combinations not estimated. N, mineral nitrogen as the only varying input; NPK, balanced mineral fertilization without exogenous carbon; N+OM, mineral nitrogen with an exogenous organic carbon source; B, biochar applied without mineral nitrogen.

Soil CO_2_ efflux increased under the regimes that supplied mineral nitrogen, whereas N_2_O and CH_4_ responded in opposite directions under the mineral and the carbon-bearing regimes (Figure 3). Soil CO_2_ efflux increased under N (+13.4%), NPK (+22.2%) and N+OM (+20.8%) (Figure 3a), and cumulative soil CO_2_ efflux under N (+22.2%) and N+OM (+29.2%) (Figure 3b). The N_2_O emission rate increased under N (+14.9%) and NPK (+42.2%) and decreased under B (−17.3%) and N+OM (−5.9%) (Figure 3c), and cumulative N_2_O emission increased under N (+18.7%) and NPK (+82.2%) (Figure 3d). Cumulative CH_4_ uptake decreased under N (−15.5%) and increased under N+OM (+9.0%) (Figure 3d).

Biochar did not change soil CO_2_ efflux or cumulative soil CO_2_ efflux (Figure 3a,b), and N+OM did not change cumulative N_2_O emission (Figure 3d). Cumulative CH_4_ uptake increased under B (+15.7%) (Table S1). The instantaneous CH_4_ uptake rate declined under N (−18.6%), matching the cumulative measure, and the methanogen marker gene *mcrA* increased under N (+27.9%) while the methanotroph marker *pmoA* did not change (Table S1).

### 2.3. Dependence of Fertilization Effects on Application Rate

**Figure 4:**
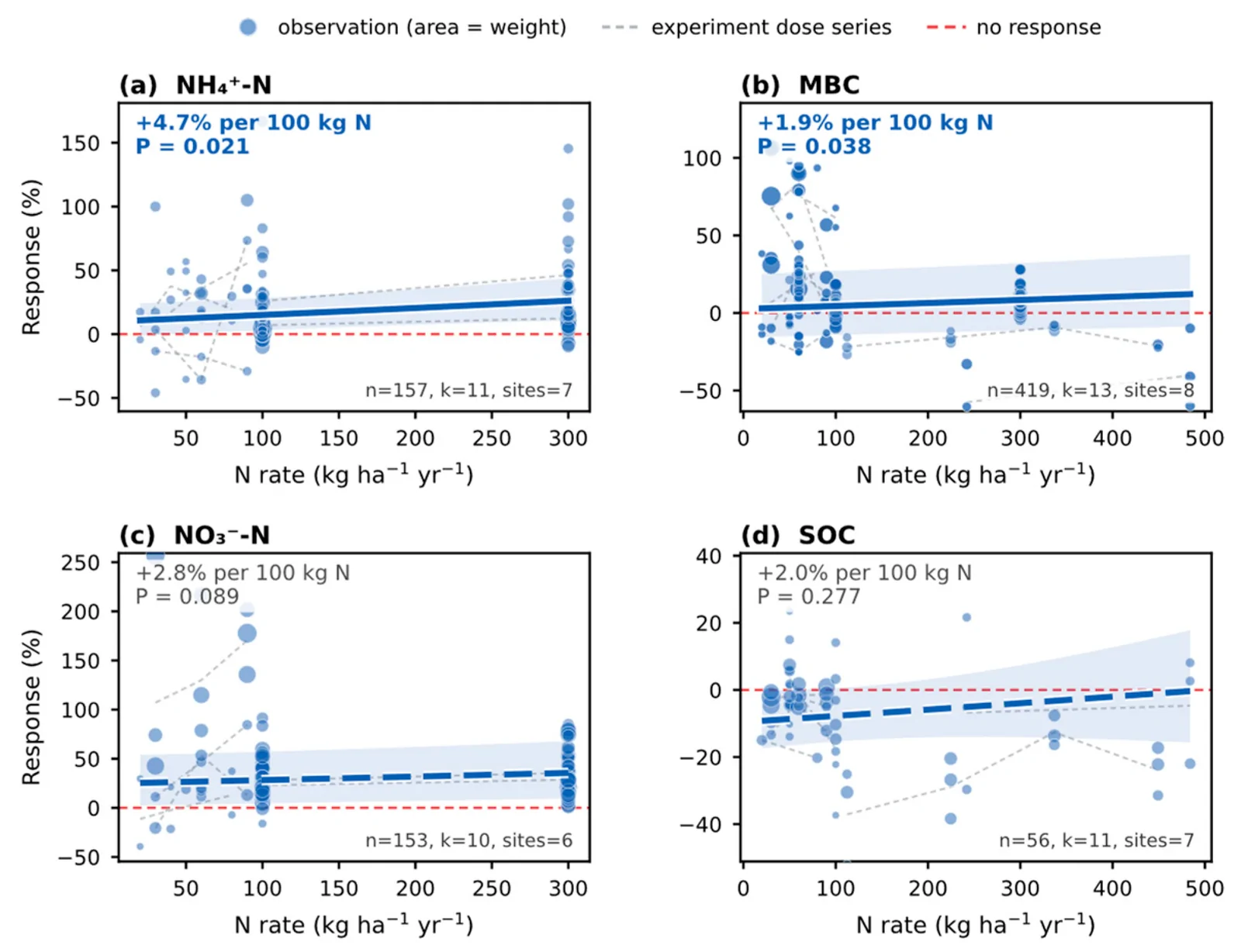
Response of the mineral nitrogen pools and the soil and microbial carbon pools to nitrogen application rate. (**a**) NH_4_^+^-N, (**b**) MBC, (**c**) NO_3_^−^-N and (**d**) SOC. Circles are paired observations scaled by their weight, and gray dashed lines join the conditions of a single experiment. The fitted meta-regression of the log response ratio (lnRR) on nitrogen application rate is drawn as a solid line where the slope differs from zero at *p* < 0.05 and as a dashed line where it does not, and the shaded band is its 95% confidence interval; the red dashed line indicates no difference from the unfertilized control. Each panel gives the change per 100 kg N ha^−1^ yr^−1^, the *p* value, and the number of paired observations (n), studies (k) and sites.

Nitrogen application rates in the experiments that support a rate model ranged from 20 to 484 kg N ha^−1^ yr^−1^, but were concentrated at the lower end of that range: the median rate was 90 kg N ha^−1^ yr^−1^ and only 22% of observations, from 8 of 37 studies, exceeded 100 kg N ha^−1^ yr^−1^. Of the tested variables with sufficient replication to support a rate model, two responded detectably to nitrogen application rate within this range (Figure 4). Ammonium nitrogen rose by 4.7% per 100 kg N ha^−1^ yr^−1^ and MBC by 1.9% (Figure 4a,b). The two pools expected to follow them did not: NO_3_^−^-N did not change with nitrogen application rate (+2.8%, *p* > 0.05), nor did SOC (+2.0%, *p* > 0.05) (Figure 4c,d).

None of the remaining soil, microbial and GHG variables changed significantly with the application rate (Figure S1). The lowest *p* values (0.06–0.10) were obtained for cumulative soil CO_2_ efflux (−8.9% per 100 kg N ha^−1^ yr^−1^), SM on the biochar axis (+2.6% per 10 t ha^−1^), soil pH (−3.8%), DOC (+2.4%) and soil CO_2_ efflux (−8.0%) (Figure S1a–e). Across the nitrogen-bearing regimes, the type of input explained more of the between-study heterogeneity than the nitrogen application rate for pH (32% vs. 0%), SOC (33% vs. 1%), TN (47% vs. 5%), NO_3_^−^-N (12% vs. 0%) and the two N_2_O measures (31% and 66% vs. ≤3%), whereas the nitrogen application rate explained more for MBC (13% vs 1%) and cumulative soil CO_2_ efflux (20% vs. 0%) (Figure S2).

### 2.4. Covariation Among the Soil, Nutrient and Microbial Responses

**Figure 5:**
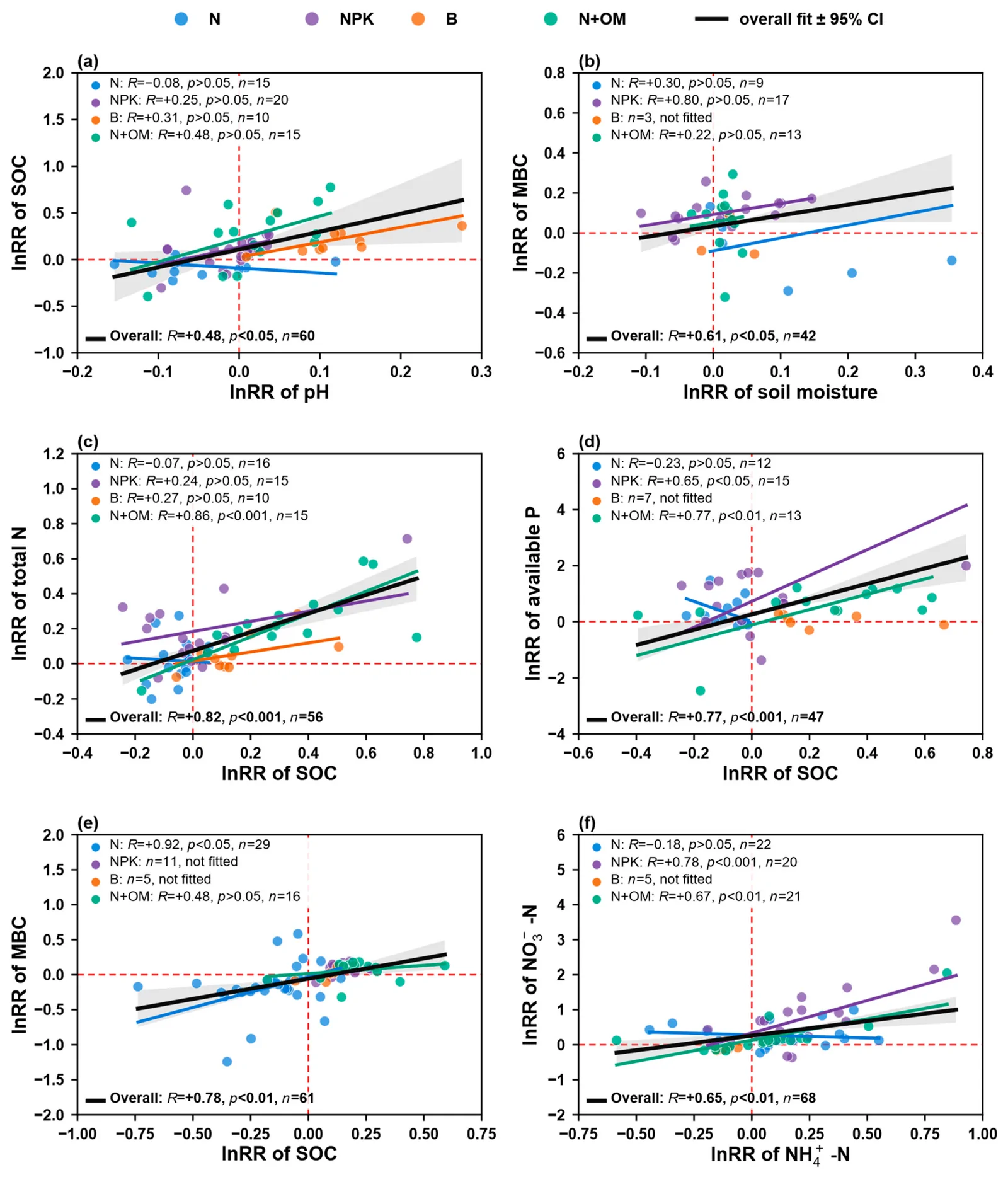
Covariation among the soil, nutrient, and microbial responses across matched treatment conditions. (**a**) SOC against pH, (**b**) MBC against SM, (**c**) TN against SOC, (**d**) AP against SOC, (**e**) MBC against SOC and (**f**) NO_3_^−^-N against NH_4_^+^-N. Axes are log response ratios, and the red dashed lines mark zero on both axes. Each point is one matched condition, colored by fertilization regime. Colored lines are the regime-specific slopes from a single model in which the four regimes share one variance estimate; a regime is fitted only where it supplied at least six matched conditions from at least four independent studies, and is otherwise shown as points alone. The black line is the overall fit and the shaded band its 95% confidence interval. Slopes are estimated within studies with a study-level random effect, and R is the corresponding partial correlation. The regimes differed only in panel (**f**) (interaction *p* = 0.008); elsewhere interaction *p* ranged from 0.270 to 0.947. N, mineral nitrogen as the only varying input; NPK, balanced mineral fertilization without exogenous carbon; N+OM, mineral nitrogen with an exogenous organic carbon source; B, biochar applied without mineral nitrogen.

The response ratios of SOC, the nutrient pools and MBC covaried across matched treatment conditions (Figure 5). Soil organic carbon increased with pH (R = 0.48), and MBC increased with SM (R = 0.61) (Figure 5a,b). Total nitrogen increased with SOC (R = 0.82), as did AP (R = 0.77) and MBC (R = 0.78) (Figure 5c–e), and NO_3_^−^-N increased with NH_4_^+^-N (R = 0.65) (Figure 5f). The covariation between the two mineral nitrogen pools differed between regimes (interaction *p* = 0.008). Nitrate nitrogen increased with NH_4_^+^-N under NPK (R = 0.78) and N+OM (R = 0.67) but not under N (Figure 5f). The remaining five covariations did not differ between regimes (interaction *p* = 0.270 to 0.947). Where a single regime was significant, it was N+OM for TN (R = 0.86, *p* < 0.001) and AP (R = 0.77, *p* < 0.01), NPK for AP (R = 0.65, *p* < 0.05) and N for MBC (R = 0.92, *p* < 0.05). Under N, TN and AP did not increase with SOC, nor SOC with pH (R = −0.23 to −0.07); the coupling of these pools was thus confined to the regimes that supply carbon and nutrients together (Figure 5).

Of the six additional couplings examined (Figure S3), five were also detectable, and none differed between regimes (interaction *p* = 0.363 to 0.695). Total phosphorus increased with pH (R = 0.68) and with SOC (R = 0.79), and AP increased with pH (R = 0.50). Ammonium nitrogen increased with SM (R = 0.77), whereas DOC declined as SM rose (R = −0.80). Microbial biomass carbon did not track DOC (R = −0.15, *p* > 0.05) (Figure S3).

## 3. Discussion

### 3.1. Soil Acidity and Soil Carbon Under Mineral Nitrogen and Carbon-Bearing Amendments

**Figure 6:**
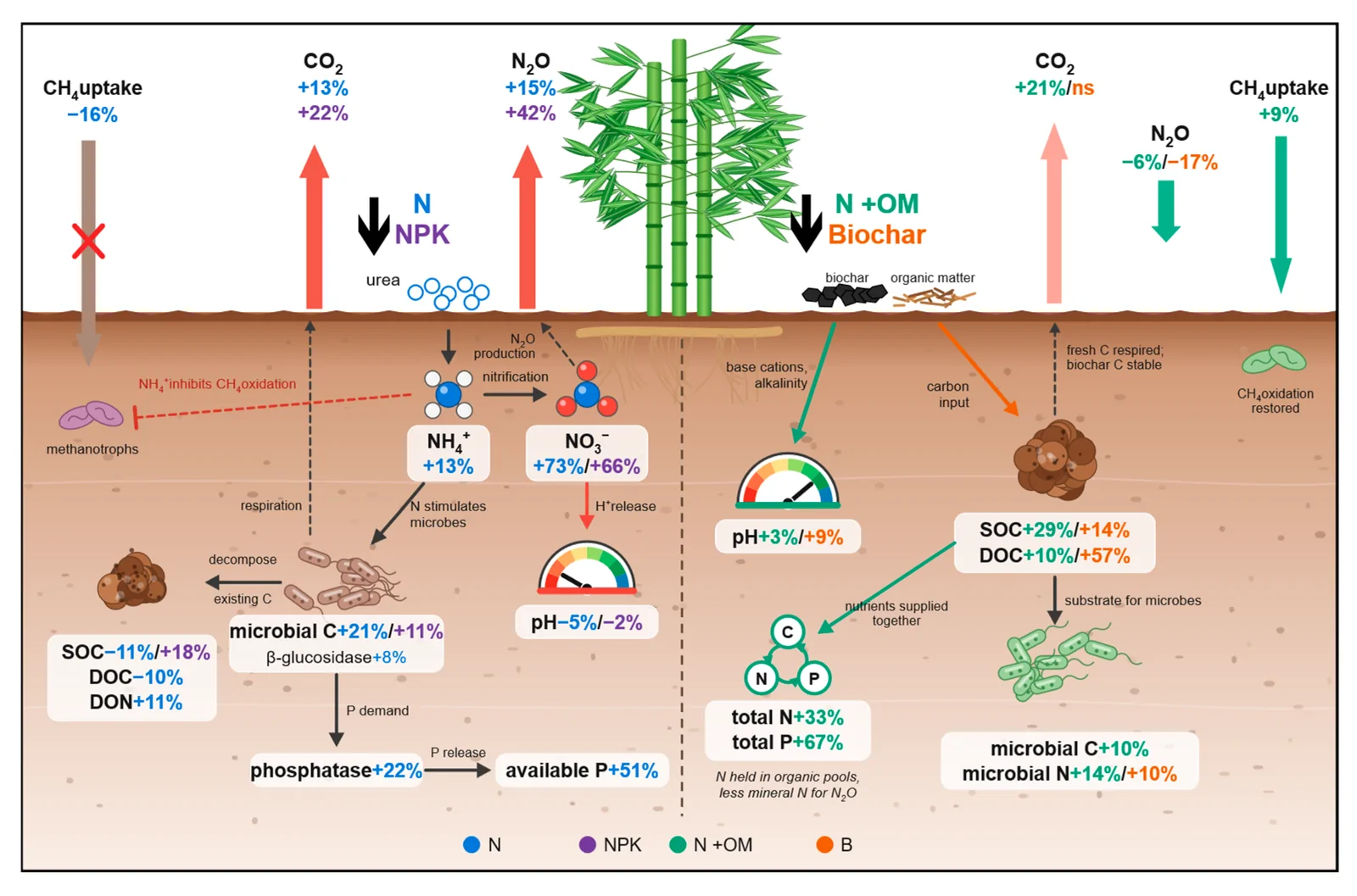
Conceptual summary of the soil and GHG responses to mineral (left: N, NPK) and carbon-bearing (right: N+OM, B) fertilization regimes in Moso bamboo forests. Values are significant mean effects (percent change relative to the unfertilized control) from Figure 1, Figure 2 and Figure 3 and Table S1, colored by regime; ns, not significant. Colored arrows show gas fluxes: upward arrows are emissions, and downward arrows are CH_4_ uptake or reduced N_2_O emission; the cross marks suppressed CH_4_ uptake. Black arrows show fertilizer inputs, thin solid arrows show soil processes, dashed arrows show gas release, and the red T-bar shows inhibition. The figure was created using BioRender: https://www.biorender.com/.

Soil pH, SOC, and DOC moved in opposite directions under N and the carbon-bearing regimes (Figure 1). The most likely mechanism is the balance between the acidity generated during nitrification of fertilizer nitrogen and the alkalinity and organic matter supplied by an amendment. The processes linking each group of regimes to the soil and GHG responses are summarized in Figure 6. Nitrification releases two H^+^ ions for every NH_4_^+^ ion oxidized, and the NO_3_^−^ formed leaches with an equivalent charge of base cations, and each application of mineral fertilizer thereby removes buffering capacity from the soil [30]; the same process has lowered soil pH measurably across Chinese croplands over three decades of mineral fertilization [31]. Biochar can reverse this effect because pyrolysis concentrates carbonates and base cations in the ash fraction [32], giving fresh biochar a liming capacity comparable to that of low-grade agricultural lime [16], and organic amendments deliver both base cations and a carbon substrate that supports microbial assimilation of NH_4_^+^, competing with nitrifiers [33]. The intermediate increase in pH under N+OM is consistent with this competition. The NPK regime, which delivers phosphorus and potassium in addition to nitrogen but carries no exogenous carbon, still lowered pH, although less than N (−1.7% against −5.3%; Figure 1a, Table S2). This is consistent with the pH increase being associated with the carbon-bearing amendments rather than with balanced nutrition, although the alkalinity and base cations that these amendments carry cannot be separated from their carbon (Section 3.6).

This mechanism is likely to matter more in Moso bamboo plantations than in many managed forests. The stands are concentrated on acidic, strongly weathered subtropical soils of low base saturation [8]; they receive high nitrogen application rates repeatedly for shoot production, and shoot harvest exports base cations from the system each year [9]. Soils already near the lower end of their buffering range are therefore likely to express the full acidifying effect of mineral nitrogen. Because acidification depends on the amount of nitrogen nitrified, whereas the pH gain under biochar depends on the alkalinity of the material, the two are not expected to be interchangeable: added alkalinity can neutralize acidity directly, whereas a lower nitrogen application rate would be expected to slow acidification rather than reverse it, which the rate data available here could not test beyond about 100 kg N ha^−1^ yr^−1^. Nitrification of fertilizer nitrogen and the export of base cations at harvest are not specific to bamboo; the contrasts between mineral nitrogen and carbon-bearing amendments reported here may therefore also apply to other intensively fertilized plantations on comparable acidic soils.

The SOC response followed the same division. Relieving microbial nitrogen limitation accelerates the mineralization of existing labile organic matter rather than building new stock [21,34], and intensively managed Moso stands receive nitrogen repeatedly and are seldom nitrogen-limited to begin with [4,6,8]. Mineral nitrogen may therefore accelerate the turnover of a carbon pool that is not being replenished, whereas the carbon-bearing regimes add to it directly; the SOC increases under N+OM and B exceeded the decrease under N. The SOC increase under NPK (+17.9%) is the exception to this division. It did not differ from the increases under the carbon-bearing regimes (Table S2). A plausible explanation is that balanced nutrition increases plant and root carbon inputs to the soil, but this could not be tested, because the phosphorus rate varies between rather than within studies. Soil organic carbon and pH also increased together across matched treatment conditions, with no significant difference between regimes (Figure 5a), and this relationship was absent under N. The covariation is consistent with both responding to the same amendment, since an amendment that carries base cations generally carries organic carbon as well, but a correlation of this kind cannot establish a common cause.

Dissolved organic carbon responded differently from SOC and indicates which carbon pool each amendment supplies. The increase under B (+56.8%) may reflect the small labile fraction of fresh biochar, which makes up only a few percent of its carbon and is decomposed within months of application [35], and would therefore represent a transient input rather than a lasting increase in SOC. Dissolved organic carbon decreased under N (−9.7%) (Figure 1d) but did not change significantly with the nitrogen application rate (Figure S1d), which is consistent with field measurements in Moso plantations, where nitrogen addition increased dissolved organic pools only where management had already altered substrate supply [36]. Dissolved organic carbon is therefore likely to be a poor indicator of whether an amendment has improved the soil carbon status of these stands, and SOC is the more appropriate measure.

### 3.2. Mineral Regimes Raise Available Nitrogen, Carbon-Bearing Regimes the Stocks

Nitrogen accumulated in different chemical forms across the two regime groups (Figure 1). A likely control is competition for NH_4_^+^ between nitrifying bacteria and the heterotrophic microbial community [33]. Because mineral fertilizer supplies NH_4_^+^ without the energy needed to assimilate it, nitrification proceeds and the added nitrogen accumulates as NO_3_^−^, the form that does not enter the organic matrix; the magnitude of the NO_3_^−^-N increase recorded here is of the same order as that reported for terrestrial ecosystems in general [10]. Where an organic carbon source accompanies the nitrogen, heterotrophic assimilation competes for the same NH_4_^+^ and directs nitrogen into microbial biomass and organic matter (Figure 6). Microbial biomass nitrogen increased under the two carbon-bearing regimes but not under the mineral regimes (Figure 2b), and TN and TP increased only under N+OM (Figure 1e,i), the single regime that supplies available nitrogen together with the energy required to assimilate it.

Two results are consistent with this partitioning depending more on the amendment’s composition than on the amount of nitrogen applied. First, SOC covaried with TN and AP across matched treatment conditions, and the relationship did not differ between regimes (Figure 5c,d), indicating that carbon and nutrient stocks increased together, as expected where they are added in the same amendment; this covariation does not by itself establish a biological link between them. Second, NO_3_^−^-N and NH_4_^+^-N covaried under NPK and N+OM but not under N (Figure 5f), and this was the only relationship in which the regimes differed. A likely explanation is that under N, NH_4_^+^ is converted to NO_3_^−^ and the NO_3_^−^ is lost, whereas balancing the nitrogen with phosphorus or with carbon slows that conversion and allows both pools to accumulate together. Biochar adds a third mechanism: sorption of NH_4_^+^, NO_3_^−^, and dissolved organics on biochar surfaces lowers their concentrations in solution and reduces leaching [37], which is consistent with the absence of any increase in NH_4_^+^-N under B (Figure 1g).

In Moso bamboo plantations, the accumulated NO_3_^−^-N therefore represents a potential loss pathway rather than a nutrient gain. These plantations occupy sloping terrain under a subtropical monsoon climate, where summer rainfall is concentrated, and percolation is rapid; NO_3_^−^ accumulated in solution is consequently likely to be leached rather than retained [38]. Mineral fertilization therefore increases the NO_3_^−^-N pool, the fraction most susceptible to leaching and denitrification, without increasing the soil nitrogen stock.

### 3.3. Microbial Biomass Varies with the Carbon Stock, Whereas Hydrolytic Enzymes Respond Weakly

Microbial biomass carbon increased under N, NPK and N+OM, whereas the hydrolytic enzymes were largely unchanged (Figure 2). Microbial biomass reflects the quantity of carbon and the physical conditions available to support it. In contrast, extracellular enzyme production is an investment that microorganisms reduce when the resource the enzyme acquires is supplied directly [39]. The two quantities are therefore not expected to move together: a larger microbial biomass accompanied by unchanged potential enzyme activity is consistent with more microbial carbon of similar specific activity. Urease and invertase did not respond to any regime, and β-glucosidase increased only under N (+7.6%); the expectation that enzyme activity declines where an amendment supplies the enzyme’s product was thus not supported by the pooled data. Differences in assay method among the primary studies may have influenced these responses but were not examined as moderators in this synthesis [40]. Nitrogen fertilization, however, supplies nitrogen but no phosphorus, which may push these soils further toward phosphorus limitation and is consistent with the 22.0% increase in phosphatase under N (Table S1, Figure 6), the response expected where nitrogen inputs accelerate phosphorus cycling [41].

Microbial biomass carbon increased with SOC and with SM across matched treatment conditions, with no difference between regimes, whereas its relationship with DOC was not detectable (Figure 5b,e and Figure S3f). Microbial biomass in these soils therefore varied with SOC and with SM rather than with DOC. This is consistent with the mulching and organic amendment practices used in intensively managed Moso stands, which add organic matter that can contribute to SOC, partly through microbial necromass [23,24]. Because SM also increased under N (+6.2%), this may explain why biomass rose under mineral nitrogen even as SOC decreased, without any gain in hydrolytic capacity. A comparable dissociation has been reported from Moso soils in which nitrogen deposition combined with intensive management raised MBC while reducing microbial diversity [4].

The β-glucosidase increase under N matches the direction of the global mean, in which nitrogen fertilization increases hydrolytic activity [15].

### 3.4. Greenhouse Gases Respond Differently to the Fertilization Regimes

The N, NPK and N+OM regimes each increased at least one of CO_2_, N_2_O, and CH_4_ (Figure 3 and Figure 6), because the three gases are produced and consumed by different processes that the same amendment affects in opposite directions. Soil CO_2_ efflux increased under N, NPK and N+OM, but not under B. Under N+OM, this reflects mineralization of the added substrate together with priming of native organic matter [34], and should be interpreted alongside the concurrent increase in SOC, because a larger efflux from a larger stock is not evidence of net carbon loss. Under N, the increase occurred without any carbon input and alongside a decrease in SOC, and may therefore indicate a net loss of soil carbon.

Nitrous oxide and CH_4_ responded in opposite directions under the mineral and the carbon-bearing regimes. Nitrous oxide production depends on the size of the mineral nitrogen pool, which the mineral regimes enlarge and which biochar reduces through sorption and through improved aeration; reductions in soil N_2_O emission under biochar of roughly half have been reported across laboratory and field treatments [18], and the lower N_2_O emission rates recorded here under B (−17.3%) and N+OM (−5.9%) are in the same direction but considerably smaller. Methane uptake depends on methanotroph activity, and NH_4_^+^ competitively inhibits methane monooxygenase, the enzyme that oxidizes CH_4_ [42], which is consistent with the lower CH_4_ uptake under mineral nitrogen and the higher uptake under N+OM (+9.0%); this mechanism has been demonstrated directly in a Moso bamboo forest [12], and the pattern is consistent with the variable direction reported for biochar effects on CH_4_ exchange [19]. Balanced mineral fertilization raised N_2_O more than N (+82.2% against +18.7% for the cumulative measure, and +42.2% against +14.9% for the emission rate) (Figure 3c,d), which is consistent with the higher nitrogen application rates in the NPK experiments that measured N_2_O (medians of about 200 and 60 kg N ha^−1^ yr^−1^ under NPK and N, respectively), with no carbon supplied to compete for the added nitrogen.

Because the gases responded differently, an assessment of a fertilization regime based on a single gas may be misleading in these plantations. The N+OM regime lowered the N_2_O emission rate and increased CH_4_ uptake while producing the largest cumulative CO_2_ increase recorded here; the N regime was unfavorable for all three gases; and biochar did not raise soil CO_2_ efflux and was associated with lower N_2_O emission and greater CH_4_ uptake (Figure 3c; Table S1), a pattern that needs confirmation in further field trials. Paired flux measurements in Moso bamboo stands have shown the same interdependence [22], and the eddy-covariance evidence that these forests are strong carbon sinks [2,5] is not sufficient on its own to establish that a given fertilization regime is climatically beneficial. These comparisons rest on fewer studies for N_2_O and CH_4_ than for CO_2_ (Section 3.6).

### 3.5. Implications for the Management of Fertilized Moso Bamboo Stands

The data indicate that soil responses depended more on the type of input than on the nitrogen application rate. Within the range that was tested, the nitrogen application rate affected only NH_4_^+^-N and MBC, and in every fit the change per 100 kg N ha^−1^ yr^−1^ was small relative to the difference between regimes (Figure 4). Soil pH illustrates this: the type of input changed pH substantially, lowering it by 5.3% under N and raising it by 9.5% under B, whereas pH did not change significantly with the nitrogen application rate (Figure S1c). Similarly, the type of input explained more of the between-study heterogeneity than the nitrogen application rate for pH, SOC and TN (Figure S2). Lowering the rate would also be expected to reduce shoot growth [43], whereas combining nitrogen with a carbon-bearing amendment was associated with higher pH and SOC than N while leaving the nitrogen supply unchanged. These results apply to nitrogen inputs of up to about 100 kg N ha^−1^ yr^−1^: because the median rate in the rate models was 90 kg N ha^−1^ yr^−1^ and only 22% of observations exceeded 100 kg N ha^−1^ yr^−1^, whether either practice would have similar effects at the higher rates used commercially cannot be established here.

Neither soil CO_2_ efflux nor cumulative soil CO_2_ efflux changed significantly with nitrogen application rate (Figure S1a,e), although both increased under N (Figure 3a,b). However, other studies have reported a dependence on rate: an optimum nitrogen application rate of 100 to 150 kg N ha^−1^ yr^−1^ has been reported globally, beyond which the soil carbon sink declined [44], and in a Moso bamboo plantation, soil CO_2_ emissions increased at 30 and 60 kg N ha^−1^ yr^−1^ but not at 90 kg N ha^−1^ yr^−1^ [22]. Because most of the experiments synthesized here applied less than 100 kg N ha^−1^ yr^−1^, they cannot show whether such an optimum applies to Moso bamboo at the higher rates used for shoot forcing [3].

### 3.6. Limitations of This Synthesis

Three limitations qualify these results. First, because each regime was compared against its own unfertilized control, the carbon supplied by organic and pyrogenic amendments is confounded with the phosphorus, potassium, base cations and alkalinity that they also carry. No experiment in this literature varied carbon while holding these properties constant, and the effects of amendment rate and of biochar properties such as feedstock and pyrolysis temperature were not tested in this synthesis. The conclusions therefore concern carbon-bearing amendments as a class rather than carbon as an isolated factor, although the SOC and MBC responses did not differ between pyrogenic and non-pyrogenic sources within N+OM (Figure S4). Second, the rate models rest on experiments applying a median of 90 kg N ha^−1^ yr^−1^, with only 22% of observations above 100 kg N ha^−1^ yr^−1^, and therefore describe responses up to moderate agronomic rates rather than at the higher rates used commercially. Third, the biochar regime and the N_2_O and CH_4_ variables rest on fewer studies than the other responses, and the N+OM gas estimates derive from biochar-based amendments.

## 4. Materials and Methods

### 4.1. Study System

Moso bamboo is a giant, monopodial, clonally spreading bamboo of subtropical East Asia. The field experiments synthesized here span 25.7–35.2° N and 105–128° E at altitudes of 11–1200 m, under a subtropical monsoon climate, with mean annual temperatures of 12.2–21.2 °C and a median annual precipitation of about 1420 mm (Table S3). The soils are predominantly acidic and strongly weathered, reported most often as Ferralsols and Ultisols with smaller numbers of red soils, Ferric Luvisols and Cambisols, soils that typically have low base saturation and limited pH buffering. Commercial stands are managed intensively: the soil is tilled, mineral nitrogen is applied annually to promote shoot emergence, and shoots and culms are harvested on a short cycle, which exports base cations from the system [3,9]. New culms arise from a connected rhizome network rather than from individual trees, which allows resources to be shared among culms within a stand.

### 4.2. Data Source and Extraction

Literature was surveyed in the Web of Science Core Collection, Scopus and the China National Knowledge Infrastructure using keywords combining (“Moso bamboo” OR “*Phyllostachys edulis*” OR “*Phyllostachys pubescens*”) AND (“fertilization” OR “nitrogen” OR “phosphorus” OR “biochar” OR “organic amendment” OR “compost” OR “straw”), with no restriction on publication year; the last search was run on 29 August 2026. After screening, 98 articles met these criteria (Table S3). Where two articles reported results from the same experiment (the same plots, treatments and unfertilized control), they were treated as a single study, which is the unit of replication in all analyses. Articles were screened by title and abstract and retained when: (a) they reported original data from field or pot experiments, reviews being excluded; (b) the study system was *Phyllostachys edulis*, and where several species were covered only the Moso bamboo data were extracted; (c) a fertilizer or amendment treatment was reported alongside a paired unfertilized control, and the amendment was identifiable in composition and, where reported, in application rate; and (d) treatment mean, control mean, a measure of dispersion and replicate number were reported or could be read from a figure. Values presented only in figures were digitized using Grafula 3 (v.2.10). Soil CO_2_ efflux was measured either by static chamber with gas chromatography or by infrared gas analyzer, in comparable numbers of studies, whereas the N_2_O and CH_4_ fluxes were measured by static chamber with gas chromatography throughout; all gas measurements come from field experiments, each compared against its own unfertilized control at the same site.

For each treatment-control contrast, we extracted the mean, standard deviation (SD) or standard error (converted to SD), and replicate number of both groups, together with the response variable and treatment as reported, the application rates of nitrogen, phosphorus, potassium, biochar, and organic amendment, the soil layer sampled, the sampling occasion, and the site name and coordinates. The compiled dataset contains 16,931 paired observations from the 98 articles; after excluding articles that tested treatments outside the four fertilization regimes, lacked a measure of dispersion, or contributed only to variables or regime–variable combinations that were not analyzed (<3 studies), 9246 observations from 69 articles entered the analyses (Text S1). An observation is the pair of means for one variable from one individual treatment on one sampling occasion at one soil layer. Observations from different treatments, including treatments compared with the same control, were treated as independent, whereas those from the same treatment on different sampling occasions were treated as non-independent and down-weighted by Equation (3). Soil respiration and CO_2_ emission rate, both measured as total soil-surface efflux, were combined as soil CO_2_ efflux. Standard deviations reported as zero or implausibly small were replaced by the median coefficient of variation in that variable.

### 4.3. Source of Variation

Treatments were assigned to four mutually exclusive fertilization regimes defined by whether an exogenous carbon source was applied and by mineral nutrient balance: N, mineral nitrogen as the only varying input; NPK, balanced mineral fertilization without exogenous carbon; N+OM, mineral nitrogen combined with an exogenous organic carbon source, namely compost, manure, rapeseed cake, straw or biochar, including biochar-based nitrogen fertilizers; and B, biochar applied without mineral nitrogen. Biochar is therefore assigned to N+OM when it is applied together with mineral nitrogen and to B when it is applied without it, so that the two regimes differ by the presence of nitrogen rather than by the amendment itself. Within N+OM, the carbon source was additionally classified as non-pyrogenic (compost, manure, rapeseed cake, straw) or pyrogenic (biochar and biochar-based fertilizers), and the two groups were compared for the variables with at least three studies in each (Figure S4). Biochar applied without mineral nitrogen was added at 5 to 40 t ha^−1^ in the experiments that reported a rate; feedstock, pyrolysis temperature and surface properties were not examined as moderators. The analyzed observations came from 66 studies (69 articles) at 21 sites: N (3348 observations, 32 studies, 10 sites), NPK (1876; 27; 13), N+OM (3228; 16; 7) and B (794; 11; 5). A regime-variable combination was analyzed only when it drew on at least three independent studies; combinations below this threshold are marked in the figures and were excluded from all estimates and contrasts, except the biochar effects on the N_2_O emission rate and cumulative CH_4_ uptake, which drew on two studies each and were not included in the between-regime tests.

### 4.4. Meta-Analysis

The log response ratio (lnRR) was used as the effect size for each observation [45]:

(1) $$\ln\left(RR\right)=\ln\left(\frac{X_{t}}{X_{c}}\right)=\ln\left(X_{t}\right)-\ln\left(X_{c}\right)$$

where *X_t_* and *X_c_* are the mean values of the variable in the treated and control groups, respectively, and the variance (v) of each lnRR was calculated as:

(2) $$v=\frac{{SD}_{t}^{2}}{n_{t}X_{t}^{2}}+\frac{{SD}_{c}^{2}}{n_{c}X_{c}^{2}}$$

where *SD_t_* and *SD_c_* are the standard deviations, and n_t_ and n_c_ are the replicate numbers of the treated and control groups. The initial weighting factor w is the inverse of v. Because a single study contributes many observations and non-independence is a recognized problem in ecological meta-analysis [46,47], the unit of analysis was shifted: an independent group was defined by the combination of study, duration, soil layer, all application rates, treatment, and response variable, giving 2125 groups, and the adjusted weight was computed as [48]:

(3) $$w^{\prime }=w/n$$

where n is the number of observations in that independence group, so that a treatment measured on many occasions receives the same total weight as one measured once. This is a standard approach for dependent effect sizes, which shifts the unit of analysis and is used where the within-study correlation among observations cannot be recovered from the primary reports [49]; it equalizes the contribution of dependent observations but does not model the correlation among them. Weighted means and 95% confidence intervals were estimated with random-effects models using the DerSimonian–Laird estimator [50]; restricted maximum likelihood was used for the two-level meta-regressions of Equation (5), where a moment estimator is not available. Standardized residuals were taken from an initial fit, and observations with an absolute value above three were removed before refitting [51,52]; 194 of the 9246 analyzed observations (2.1%) were excluded in this way. Mean effects were converted to percent change:

(4) $$\left.Change\;\left(\%\right)=\left(e^{ln\left(RR\right)}-1\right.\right)\times 100\%.$$

where positive and negative values indicate an increase and a decrease relative to the unfertilized control. For CH_4_, uptake is expressed as a positive quantity; a positive change therefore denotes a stronger soil CH_4_ sink. An effect was considered significant when its 95% confidence interval did not overlap zero (*p* < 0.05). We further tested for potential publication bias using Rosenberg fail-safe numbers [53]. Funnel asymmetry was tested with an Egger-type regression [54]. Fail-safe numbers exceeded 5n + 10, where n is the number of observations before outlier screening, for 37 of the 39 significant effects, the exceptions being DOC under N and MBN under B, and funnel asymmetry was indicated for DOC, NO_3_^−^-N, MBN, cumulative soil CO_2_ efflux and the N_2_O emission rate (Text S2, Table S4a,b). The sensitivity of the pooled effects to the outlier screen, the heterogeneity estimator and the exclusion of pot experiments and laboratory incubations was also examined (Text S3, Table S5). For each variable, the total heterogeneity (Q_T_) was partitioned into between-group heterogeneity (Q_B_), attributed to the fertilization regime, and within-group heterogeneity (Q_W_). A significant Q_B_ statistic (*p* < 0.05) indicates that the regime explains a significant portion of the heterogeneity (Table S2). The share of between-study heterogeneity explained by regime and by nitrogen application rate was also compared (Figure S2).

Rate dependence and covariation were both tested with two-level random-effects meta-regression, with a study random intercept fitted on top of the known sampling variance of each observation. For covariation, the moderator was additionally split into a study mean and a within-study deviation [55]:

(5) $${lnRR}_{ij}=\beta_{0}+\beta_{1}(x_{ij}-{\bar{x}}_{i})+\beta_{2}{\bar{x}}_{i}+u_{i}+e_{ij}+\varepsilon_{ij}$$

where lnRR_ij_ is the effect size of observation j in study i, x_ij_ the moderator and ${\bar{x}}_{i}$ its mean in study i; β_1_ is the within-study slope reported here and β_2_ the between-study slope; u_i_ is the study random intercept, e_ij_ the residual heterogeneity and ε_ij_ the sampling error with known variance v_ij_. Models were fitted by restricted maximum likelihood, a specification equivalent to *rma.mv* in metafor [52]. Coefficients were tested against zero, and their 95% confidence intervals were constructed using a t-distribution with degrees of freedom equal to the number of independent studies contributing to the fit minus the number of fitted coefficients. The study was treated as the unit of independence here, as in the weighting of the effect sizes (Equation (3)), because observations within a study share soil, climate and management and are not independent replicates.

Fertilizer application rate dependence was tested by meta-regression of lnRR on application rate, separately for the nitrogen and the biochar axis. The variable was analyzed only where at least three studies had applied more than one application rate. Slopes are given per 100 kg N ha^−1^ yr^−1^ or per 10 t biochar ha^−1^. Nitrogen application rates above 600 kg N ha^−1^ yr^−1^ were not included in the rate models. The nitrogen application rates represented in these models ranged from 20 to 484 kg N ha^−1^ yr^−1^, with a median of 90 kg N ha^−1^ yr^−1^; the rate results thus describe the range from nitrogen deposition to moderate agronomic application rather than the rates used for shoot forcing in commercial stands.

Covariation was tested by regressing the lnRR of one variable on that of another across treatment conditions matched by study, fertilizer treatment, soil layer, application rate and sampling occasion, with the predictor decomposed as above. Two models were fitted per pair, an overall model and one allowing the within-study slope to differ among regimes, tested by a joint Wald test; R is the partial correlation implied by the same model. Pairs required at least fifteen matched conditions from eight studies, and a regime slope at least six conditions from four studies. Analyses were performed in Python 3.11, and figures were generated in Matplotlib 3.10.9.

## 5. Conclusions

Soil acidity and soil carbon in Moso bamboo plantations responded in opposite directions to mineral nitrogen and to carbon-bearing amendments. Nitrogen applied alone acidified the soil, reduced SOC and increased the readily available inorganic nitrogen pool. The carbon-bearing regimes raised pH, increased SOC and, under N+OM, TN, and retained nitrogen in organic and microbial pools. Balanced NPK fertilization also raised SOC but still lowered pH. Microbial biomass carbon increased under N, NPK and N+OM and is therefore of limited value as an indicator of function on its own, while hydrolytic enzyme activities were largely unchanged. The N, NPK and N+OM regimes each increased at least one of CO_2_, N_2_O and CH_4_, and none of them reduced all three gases simultaneously. Within the range of rates represented here, mostly below 100 kg N ha^−1^ yr^−1^, the nitrogen application rate altered only NH_4_^+^-N and MBC detectably, and by less than the difference between regimes. Soil organic carbon and the nutrient pools covaried across treatment conditions where carbon or balanced nutrients were supplied, but not under N, which is consistent with these variables responding jointly to soil carbon status only when the amendment increases it. Because the carbon in these amendments is applied together with their phosphorus, potassium, base cations and alkalinity, these conclusions concern carbon-bearing amendments as a class rather than carbon as an isolated factor.

This synthesis supports four practices for fertilized Moso bamboo stands. First, at nitrogen inputs of up to about 100 kg N ha^−1^ yr^−1^, which most experiments applied, mineral nitrogen should be combined with a carbon-bearing amendment rather than applied alone: N+OM raised soil pH by 3.4% and SOC by 28.7%, whereas N lowered them by 5.3% and 11.4%, and N+OM increased TN without the NO_3_^−^-N accumulation that exposes mineral nitrogen to leaching on sloping stands. Second, biochar is a candidate for restoring acidified stands, because at the 5–40 t ha^−1^ applied in these experiments it raised pH by 9.5% and SOC by 14.0% without increasing soil CO_2_ efflux, although this evidence comes from 11 studies at five sites. Third, where balanced NPK fertilizer is used, its weaker acidifying effect should be weighed against the largest N_2_O increases recorded here. Fourth, the effect of a practice on soil carbon is better monitored with SOC and pH than with DOC, which may largely reflect transient inputs, or with MBC, which increased even where SOC declined. Because N+OM lowered the N_2_O emission rate and increased CH_4_ uptake but produced the largest cumulative CO_2_ increase, fertilization practices should be evaluated against all three gases. Whether these practices hold at the higher rates used for shoot production, and whether lowering the rate would bring similar benefits, cannot be judged from the present data; long-term field trials that cross nitrogen application rate with carbon-bearing amendments and measure soil properties and all three gases at the same sites are needed to resolve this.

## Data Availability

The dataset compiled for this meta-analysis is openly available in Figshare at https://doi.org/10.6084/m9.figshare.33944131.

## Supplementary Materials

The following supporting information can be downloaded at https://www.mdpi.com/article/10.3390/plants15213301/s1, Figure S1: Response of the remaining soil, microbial and greenhouse gas variables to fertilizer application rate; Figure S2: Share of the between-study heterogeneity explained by the type of input (fertilization regime) and by the nitrogen application rate; Figure S3: Covariation among the additional soil and nutrient responses across matched treatment conditions; Figure S4: Response of the soil carbon pools to nitrogen applied with organic matter, separated by the form of the carbon added; Table S1: Response of the additional soil, microbial and gas variables that met the three-study threshold in at least one fertilization regime; Table S2: The *p* value of the Q_B_ between-group heterogeneity test comparing the soil, microbial and gas variables across fertilization regimes; Table S3: Climatic and geographic information of the 98 articles (94 studies) included in this meta-analysis; Table S4a: Publication bias: Rosenberg weighted fail-safe numbers for every fertilization regime × variable combination presented in Figure 1, Figure 2 and Figure 3 that met the three-study threshold; Table S4b: Egger-type test for funnel asymmetry for each variable; Table S5: Sensitivity of the pooled effects to the analytical choices; Text S1: Selection of articles and data used in the analyses; Text S2: Publication bias tests; Text S3: Sensitivity analyses. References [56,57,58,59,60,61,62,63,64,65,66,67,68,69,70,71,72,73,74,75,76,77,78,79,80,81,82,83,84,85,86,87,88,89,90,91,92,93,94,95,96,97,98,99,100,101,102,103,104,105,106,107,108,109,110,111,112,113,114,115,116,117,118,119,120,121,122,123,124,125,126,127,128,129,130,131,132,133,134,135,136,137,138,139,140,141,142] are cited in the Supplementary Materials (Table S3).

## Abbreviations

The following abbreviations are used in this manuscript:

AP	Available phosphorus
B	Biochar applied without mineral nitrogen (fertilization regime)
CH_4_	Methane
C:N	Carbon-to-nitrogen ratio
CO_2_	Carbon dioxide
DOC	Dissolved organic carbon
DON	Dissolved organic nitrogen
GHG	Greenhouse gas
lnRR	Log response ratio
MBC	Microbial biomass carbon
MBN	Microbial biomass nitrogen
N	Mineral nitrogen alone (fertilization regime)
N+OM	Mineral nitrogen combined with an exogenous organic carbon source (fertilization regime)
N_2_O	Nitrous oxide
NH_4_^+^-N	Ammonium nitrogen
NO_3_^−^-N	Nitrate nitrogen
NPK	Balanced mineral fertilization supplying nitrogen, phosphorus and potassium (fertilization regime)
Q_B_	Between-group heterogeneity
Q_T_	Total heterogeneity
Q_W_	Within-group heterogeneity
SD	Standard deviation
SM	Soil moisture
SOC	Soil organic carbon
TN	Total nitrogen
TP	Total phosphorus
