# Supplementary material for: Soil Responses to Mineral Nitrogen and Carbon-Bearing Amendments in Moso Bamboo Forests: A Meta-Analysis
Source: Plants (Basel). 2026 Oct 26;15(21):3301. doi: 10.3390/plants15213301 (PMC13765591; doi:10.3390/plants15213301)
Supplement: Supplementary file 1 [file plants-15-03301-s001.zip › plants-4631726-supplementary.pdf]

Supporting information

Soil Responses to Mineral Nitrogen and Carbon-Bearing Amendments in Moso Bamboo Forests: A Meta-Analysis

Amina Qamar[1], Nasir Shad[2], Abbas Ali[3], Nizar Nawaz[4], Chunce Guo[1]*

[1] Jiangxi Provincial Key Laboratory of Improved Variety Breeding and Efficient Utilization of Native Tree Species, College of Forestry, Jiangxi Agricultural University, Nanchang 330045, China; aminaqamar774@gmail.com, chunceguo@jxau.edu.cn

[2] School of Ecology and Applied Meteorology, Nanjing University of Information Science & Technology, Nanjing 210044, China; 202351530001@nuist.edu.cn, dearbotanist@gmail.com

[3] Key Laboratory of National Forestry and Grassland Administration on Forest Ecosystem Protection and Restoration of Poyang Lake Watershed, Jiangxi Agricultural University, Nanchang 330045, China; jainkhan619@gmail.com

[4] Jiangxi Provincial Key Laboratory of Subtropical Forest Resources Cultivation, College of Forestry, Jiangxi Agricultural University, Nanchang 330045, China; nizarnawaz66@gmail.com

* Correspondence: Chunce Guo (chunceguo@jxau.edu.cn), Phone (+86-13521998505)

Text S1. Selection of articles and data used in the analyses. The literature search (Section 4.2) yielded 98 articles that met the inclusion criteria; their sites and climate are listed in Table S3. Where two articles reported results from the same experiment, that is, the same plots, treatments and unfertilized control, they were treated as a single study, which is the unit of replication in all analyses. This applied to four pairs of articles, Cheng et al. (2025a, 2025b), Huang et al. (2025a, 2026), Jiang et al. (2025a, 2026a) and Yang et al. (2025a, 2025c), so that the 98 articles describe 94 studies. Data were extracted from all 98 articles, giving 16,931 paired observations. Of these, 2,455 observations did not enter the four-regime analysis, namely 2,442 from treatments outside the four fertilization regimes, such as silicon, phosphorus-only or biofertilizer treatments, and 13 laboratory measurements of gas release; a further 334 lacked a measure of dispersion, which left 14,142 observations with a computable sampling variance. Among them, 4,896 paired observations belonged to variables not analyzed in this synthesis, such as plant traits, microbial community composition, nutrient leaching and soil gas concentrations, or to regime–variable combinations represented by fewer than three studies. At the article level, 12 articles reported only treatments outside the four regimes, four reported no measure of dispersion, and 13 contributed only to variables or combinations that were not analyzed. The remaining 69 articles (66 studies at 21 sites) contributed 9,246 paired observations to the analyses before the outlier screen: 8,108 to the 70 regime–variable estimates in Figures 1–3, 1,016 to the estimates in Table S1, and 122 to the biochar N_2O emission rate (Figure 3c). By regime, N contributed 3,348 observations (32 studies, 10 sites), NPK 1,876 (27; 13), N+OM 3,228 (16; 7) and B 794 (11; 5). The outlier screen (Main manuscript; Section 4.4) then removed 194 observations (2.1%), namely 175 from Figures 1–3, 17 from Table S1 and 2 from the biochar N_2O emission rate; the numbers of observations shown in the figures and tables are those retained after screening. Articles that did not contribute data to the analyses are marked ‡ in Table S3.

Text S2. Publication bias tests. Rosenberg's weighted fail-safe number (Rosenberg, 2005) was calculated for each fertilization regime × variable combination in Figures 1–3 that met the three-study threshold, and an effect was considered robust when it exceeded $5n + 10$, where n is the number of observations. Funnel asymmetry was tested for each variable with an Egger-type regression of lnRR on its standard error (Egger et al., 1997), with fertilization regime as a moderator and a study random intercept. Of the 39 effects that differed significantly from zero, 37 exceeded the fail-safe threshold (Table S4a; the exceptions were DOC under N and MBN under B), and funnel asymmetry was indicated for five variables (Table S4b).

Text S3. Sensitivity analyses. The 70 pooled effects in Figures 1–3 that met the three-study threshold were refitted without removing outlying observations, with the between-study variance estimated by restricted maximum likelihood (REML), and with pot experiments and laboratory incubations excluded (Table S5). The direction and significance of 65, 66 and 68 of the 70 estimates, respectively, were unchanged. Without outlier removal, SOC under N and the N_2O emission rate under N+OM were no longer significant, β-glucosidase under N changed sign, and SM under B and NO_3^--N under N+OM became significant; REML gave identical point estimates. The contrasts of N with N+OM and with B for soil pH and SOC also remained significant ($P < 0.001$) with standard errors clustered by study.

Table S1. Response of the additional soil, microbial and gas variables that met the three-study threshold in at least one fertilization regime. Change is the percent difference from the unfertilized control with its 95% confidence interval; n is the number of paired observations retained after outlier screening. The biochar effect on cumulative CH_4 uptake is based on two studies.

Variable	Regime	Change (%)	95% CI lower	95% CI upper	P	n
Soil C:N ratio	N	-1.3	-8.6	+6.6	0.743	10
	N+OM	+2.6	-3.7	+9.2	0.429	16
	NPK	-6.2	-20.8	+11.1	0.461	13
Bulk density	NPK	+1.4	-1.9	+4.7	0.410	61
SOC stock	NPK	-1.7	-11.6	+9.4	0.757	24
Phytolith-occluded carbon	NPK	-5.1	-26.7	+22.8	0.690	25
Alkali-hydrolyzable nitrogen	N+OM	+38.1	+19.4	+59.7	<0.001	15
	NPK	+15.1	-1.3	+34.2	0.072	57
Available potassium	NPK	-2.6	-17.6	+15.1	0.754	52
Total potassium	NPK	+5.2	+1.2	+9.2	0.010	33
Microbial biomass phosphorus	N	+44.0	-13.7	+140.1	0.163	20
Phosphatase	N	+22.0	+7.6	+38.3	0.002	33
	NPK	+12.5	-12.9	+45.3	0.366	40
Catalase	NPK	-2.2	-35.5	+48.2	0.915	36
mcrA gene abundance	N	+27.9	+0.1	+63.4	0.049	77
pmoA gene abundance	N	+0.4	-27.6	+39.2	0.981	78
Heterotrophic respiration	N+OM	+3.8	-0.8	+8.7	0.109	78
CH_4 uptake rate	N	-18.6	-24.4	-12.4	<0.001	326
Cumulative CH_4 uptake (2 studies)	B	+15.7	+2.8	+30.2	0.015	5

Table S2. The P value of the Q_B between-group heterogeneity test comparing the soil, microbial and gas variables across fertilization regimes. Q_B is the between-group Q statistic testing whether the fertilization regimes differ from one another in their effect on each variable, with its P value. Significant results are in bold. Soil respiration and CO_2 emission rate are combined as soil CO_2 efflux (Section 4.2). A dash marks a pair in which one regime had fewer than three studies.

Variable	N vs NPK	N vs N+OM	N vs B	NPK vs N+OM	NPK vs B	N+OM vs B
pH	**0.001**	**<0.001**	**<0.001**	**<0.001**	**<0.001**	**0.024**
Soil CO_2 efflux	0.108	0.100	0.227	0.789	**0.036**	0.054
SM	**<0.001**	0.530	0.159	**<0.001**	**0.011**	0.416
SOC	**<0.001**	**<0.001**	**<0.001**	0.254	0.774	0.239
DON	0.642	**<0.001**	—	0.530	—	—
TN	**0.035**	**<0.001**	**0.038**	**0.007**	0.722	**0.012**
DOC	**<0.001**	**<0.001**	**<0.001**	**<0.001**	**<0.001**	**<0.001**
NH_4^+-N	0.961	**<0.001**	**0.001**	0.250	0.554	0.113
NO_3^--N	0.689	**<0.001**	—	**<0.001**	—	—
AP	0.062	0.253	0.187	0.817	0.150	0.560
TP	0.799	**0.011**	0.433	**0.030**	0.905	**0.029**
MBC	0.154	**0.021**	0.354	0.763	0.552	0.477
MBN	0.384	**0.020**	0.210	0.359	0.829	0.664
Urease	0.395	0.278	—	0.834	—	—
β-glucosidase	0.980	0.508	—	0.698	—	—
Invertase	0.078	0.441	—	0.075	—	—
Cumulative soil CO_2 efflux	—	0.350	0.399	—	—	0.203
N_2O emission rate	**0.002**	**<0.001**	—	**<0.001**	—	—
Cumulative N_2O emission	**0.013**	**0.049**	—	**0.001**	—	—
Cumulative CH_4 uptake	—	**<0.001**	—	—	—	—
Additional variables reported in Table S1						
Soil C:N ratio	0.636	0.469	—	0.218	—	—
Alkali-hydrolyzable nitrogen	—	—	—	0.096	—	—
Phosphatase	0.592	—	—	—	—	—

Note: omnibus Q_B test across all regimes with at least three studies: DOC, Q_B = 74.1 (df = 3, P < 0.001); pH, Q_B = 73.8 (df = 3, P < 0.001); SOC, Q_B = 54.0 (df = 3, P < 0.001); N_2O emission rate, Q_B = 47.7 (df = 2, P < 0.001); NO_3^--N, Q_B = 44.8 (df = 2, P < 0.001); cumulative CH_4 uptake, Q_B = 24.6 (df = 1, P < 0.001); SM, Q_B = 29.8 (df = 3, P < 0.001); TN, Q_B = 18.7 (df = 3, P < 0.001); cumulative N_2O emission, Q_B = 14.7 (df = 2, P < 0.001); TP, Q_B = 12.7 (df = 3, P = 0.005); DON, Q_B = 5.9 (df = 2, P = 0.052); MBC, Q_B = 7.5 (df = 3, P = 0.057); soil CO_2 efflux, Q_B = 7.0 (df = 3, P = 0.073); NH_4^+-N, Q_B = 6.9 (df = 3, P = 0.076); invertase, Q_B = 3.7 (df = 2, P = 0.160); AP, Q_B = 3.8 (df = 3, P = 0.286); MBN, Q_B = 3.3 (df = 3, P = 0.354); cumulative soil CO_2 efflux, Q_B = 2.0 (df = 2, P = 0.375); urease, Q_B = 1.0 (df = 2, P = 0.606); β-glucosidase, Q_B = 0.3 (df = 2, P = 0.880).

Table S3. Climatic and geographic information of the 98 articles (94 studies) included in this meta-analysis. Articles marked ‡ did not contribute data to the analyses (Text S1).

Notes: values were taken from the site description of each source article. Coordinates are given in decimal degrees (north latitude, east longitude); altitude, temperature and precipitation are single values or the range the article reports. A dash marks a field for which no value was reported by any article describing that site. Where articles describing the same site at identical coordinates gave different descriptions, the most frequently reported value was used throughout, and the soil was named in the World Reference Base system wherever any article did so, so that each site carries one description. Long., longitude; Lat., latitude; Alt., altitude; Temp, mean annual temperature; Pre, mean annual precipitation.

Articles	Location	Long.	Lat.	Alt. (m)	Climate	Soil	Temp (°C)	Pre (mm)
Cao et al. (2020)	Suichang, Zhejiang	119.17	28.05	305	Subtropical monsoon	Ferralsol	16.8	1510
Chen et al. (2019)	Sanren, Suichang, Zhejiang	119.17	28.57	305	Subtropical monsoon	Ferralsol	16.8	1510
Chen et al. (2024)	Lin'an, Hangzhou, Zhejiang	119.84	30.38	85	Subtropical humid monsoon	Ferralsol	17.1	1250–1500
Chen et al. (2025)	Daiyun Mountain, Dehua, Fujian	118.08	25.72	–	Subtropical monsoon	Ultisol	20.0	1800
Chen et al. (2026a)	Anji, Zhejiang	119.63	30.48	235–238	Subtropical monsoon	Ferralsol	16.1	1423
Chen et al. (2026b)‡	Sanren, Suichang, Lishui, Zhejiang	118.68	28.22	–	Mid-subtropical monsoon	Yellow soil	16.8	1510
Cheng et al. (2025a)	Lin'an, Hangzhou, Zhejiang	119.83	30.23	–	Subtropical monsoon	Ferralsol	15.6	1614
Cheng et al. (2025b)	Lin'an, Hangzhou, Zhejiang	119.83	30.23	–	Subtropical monsoon	Ferralsol	15.6	1614
Chu et al. (2024a)	Hushi, Chishui, Guizhou	106.10	28.50	–	Subtropical humid monsoon	Purple-yellow sandy loam	18.1	1196
Chu et al. (2024b)	Chishui, Guizhou	105.90	28.38	–	Subtropical humid monsoon	Purple-yellow sandy loam	18.1	1196
Deng et al. (2020)	Yuhang, Hangzhou, Zhejiang	119.67	30.15	–	Subtropical maritime monsoon	Ultisol	17.6	1446
Gao et al. (2016)‡	Miaogao, Suichang, Zhejiang	119.17	28.57	305	Subtropical monsoon	Ferralsol	16.8	1510
Gao et al. (2026)‡	Qianjiangyuan Forest Ecosystem Research Station, Zhejiang	119.95	29.47	–	Greenhouse / pot experiment	Yellow loam	–	–
Ge et al. (2019)	Qianjiangyuan Forest Ecological Research Station, Zhejiang	119.93	30.05	11–536	Subtropical humid monsoon	Haplic Luvisol	16.1	1442
Ge et al. (2020)	Miaoshanwu National Reserve, Zhejiang	119.93	30.05	11–536	Subtropical humid monsoon	Haplic Luvisol	16.1	1442

Articles	Location	Long.	Lat.	Alt. (m)	Climate	Soil	Temp (°C)	Pre (mm)
He et al. (2023)	Zhejiang A&F University	119.72	30.38	–	Greenhouse / pot experiment	Ferralsol	–	–
He et al. (2026)‡	Lin'an, Hangzhou, Zhejiang	119.78	30.22	120	Subtropical monsoon	Ferralsol	15.6	1420
Huang et al. (2019)	Qingshan Township, Lin'an, Zhejiang	119.37	30.32	100–150	Mid-subtropical monsoon	Ferralsol	15.8	1422
Huang et al. (2020)	Qingshan, Hangzhou, Zhejiang	119.37	30.32	100–150	Mid-subtropical monsoon	Ferralsol	15.8	1422
Huang et al. (2021)	Shankou-Township	119.70	30.23	100–300	Subtropical monsoon	Ferralsol	15.6	1420
Huang et al. (2025a)‡	Xiaofeng, Anji, Zhejiang	119.47	30.63	–	Mid-subtropical monsoon	Ferric Luvisol	17.0	1300
Huang et al. (2025b)	Chishui, Guizhou	105.60	28.25	–	Subtropical humid monsoon	Ultisol	18.1	1196
Huang et al. (2026)‡	Xiaofeng, Anji, Zhejiang	119.47	30.63	–	Mid-subtropical monsoon	Ferric Luvisol	17.0	1300
Jiang et al. (2025a)	Anji, Zhejiang	119.63	30.48	235–238	Subtropical monsoon	Ferralsol	16.1	1423
Jiang et al. (2025b)	Nimacun, Hangzhou, Zhejiang	119.68	30.32	150	Mid-subtropical monsoon	Ultisol	17.8	1564
Jiang et al. (2025c)	Qingshan, Zhejiang	118.85	29.93	150–200	Subtropical monsoon	Ferralsol	16.0	1450
Jiang et al. (2026a)	Anji, Zhejiang	119.63	30.48	235–238	Subtropical monsoon	Ferralsol	16.1	1423
Jiang et al. (2026b)‡	Langbi, Hangzhou, Zhejiang	119.78	30.22	120	Subtropical monsoon	Ferralsol	15.6	1420
Jiang et al. (2026c)‡	Langbi, Hangzhou, Zhejiang	119.78	30.22	120	Subtropical monsoon	Ferralsol	15.6	1420
Jin et al. (2011)	Shangyang Township, Longquan, Zhejiang	118.88	27.89	–	–	–	17.5	1450
Kim et al. (2018)‡	Jinju	128.10	35.15	50–67	Subtropical monsoon	Inceptisol	13.1	1512
Lei et al. (2017)	Qingshan, Hangzhou, Zhejiang	119.70	30.23	100–300	Subtropical monsoon	Ferralsol	15.6	1420
Li et al. (2013)	Anji County, northwest, Zhejiang	119.65	30.48	150	Subtropical monsoon	Ferralsol	15.3	1422
Li et al. (2016)	Qingshan Township, Lin'an, Zhejiang	119.70	30.23	100–300	Subtropical monsoon	Ferralsol	15.6	1420
Li et al. (2018a)	Linan, Zhejiang	119.75	30.17	90–200	Subtropical humid monsoon	Ferralsol	15.9	1350–1500
Li et al. (2018b)	Qingshan, Hangzhou, Zhejiang	119.70	30.23	100–300	Subtropical monsoon	Ferralsol	15.6	1420

Articles	Location	Long.	Lat.	Alt. (m)	Climate	Soil	Temp (°C)	Pre (mm)
Li et al. (2019)	Qingshan Township, Hangzhou, Zhejiang	119.70	30.23	100–300	Subtropical monsoon	Ferralsol	15.6	1420
Li et al. (2020a)	Xixi National Wetland Park, Hangzhou, Zhejiang	120.05	30.25	–	Subtropical monsoon (marginal)	Paddy soil	16.4	1100–1600
Li et al. (2020b)	Putao Jie, Changxing, Zhejiang	119.75	30.97	–	Subtropical monsoon	Ferralsol	15.6	1309
Li et al. (2021a)	Qingshan, Hangzhou, Zhejiang	119.70	30.23	100–300	Subtropical monsoon	Ferralsol	15.6	1420
Li et al. (2021b)	Qingshan, Hangzhou, Zhejiang	119.70	30.23	100–300	Subtropical monsoon	Ferralsol	15.6	1420
Li et al. (2022a)	Qingshan Township, Lin'an, Hangzhou, Zhejiang	119.70	30.23	100–300	Subtropical monsoon	Ferralsol	15.6	1420
Li et al. (2022b)	Qingshan, Hangzhou, Zhejiang	119.70	30.23	100–300	Subtropical monsoon	Ferralsol	15.6	1420
Li et al. (2024a)	Qingshan, Hangzhou, Zhejiang	119.70	30.23	100–300	Subtropical monsoon	Ferralsol	15.6	1420
Li et al. (2024b)	Qingshan Township, Hangzhou	119.78	30.22	120	Subtropical monsoon	Ferralsol	15.6	1420
Li et al. (2024c)	Yixing City, Wuxi, Jiangsu	119.75	31.25	102	Subtropical monsoon	–	15.8	1184
Li et al. (2025)‡	Tianhuanping, Anji, Zhejiang	119.70	30.48	300–380	Subtropical monsoon	Ferric Luvisol	16.2	1300
Liu et al. (2011)	Qingshan Town, Zhejiang	119.78	30.37	–	Subtropical humid monsoon	Ferralsol	15.6	1420
Liu et al. (2023a)	Heping, Changxing, Zhejiang	119.91	30.79	–	–	–	15.6	1309
Liu et al. (2023b)‡	Fuzhou, Lin'an	119.27	26.15	–	Greenhouse / pot experiment	–	–	–
Liu et al. (2023c)‡	–	119.29	26.15	–	–	–	15.6	1309
Luan et al. (2022)	Changning County, Sichuan	105.02	28.45	260–1000	Subtropical humid monsoon	Cambisol	18.3	1142
Lv et al. (2020)	Hangzhou	119.75	30.17	90–200	Subtropical humid monsoon	Ferralsol	15.9	1350–1500
Lv et al. (2022)‡	Qingshan, Hangzhou, Zhejiang	119.70	30.23	100–300	Subtropical monsoon	Ferralsol	15.6	1420
Ni et al. (2021)	Anji, Zhejiang	119.62	30.38	200–500	Subtropical monsoon	Luvisol	16.0	1300
Peng et al. (2019)	Qingshan, Hangzhou, Zhejiang	119.70	30.23	100–300	Subtropical monsoon	Ferralsol	15.6	1420

Articles	Location	Long.	Lat.	Alt. (m)	Climate	Soil	Temp (°C)	Pre (mm)
Qin et al. (2017)	Sanren town, Suichang, Zhejiang	119.17	28.57	305	Subtropical monsoon	Ferralsol	16.8	1510
Song et al. (2015)	Qingshan Town, Zhejiang	119.70	30.23	100–300	Subtropical monsoon	Ferralsol	15.6	1420
Song et al. (2020)‡	Qingshan, Hangzhou, Zhejiang	119.70	30.23	100–300	Subtropical monsoon	Ferralsol	15.6	1420
Su et al. (2019)‡	Huangshan	118.53	32.20	492	Mid-subtropical humid monsoon	Ultisol	15.3	1500
Su et al. (2026)‡	Changning, Sichuan	105.02	28.45	260–1000	Subtropical humid monsoon	Cambisol	18.3	1142
Sun et al. (2025)‡	Langbi, Hangzhou, Zhejiang	119.78	30.22	120	Subtropical monsoon	Ferralsol	15.6	1420
Wang et al. (2021)	Changning, Sichuan	105.02	28.45	260–1000	Subtropical humid monsoon	Cambisol	18.3	1142
Wang et al. (2026a)	Hangzhou, Zhejiang	119.78	30.22	120	Subtropical monsoon	Ferralsol	15.6	1420
Wang et al. (2026b)‡	Wuxie National Forest Park, Zhejiang	120.05	29.72	220	Mid-subtropical monsoon	Red and yellow soil	17.6	1298
Xie et al. (2026)‡	Hangzhou, Zhejiang	119.78	30.22	120	Subtropical monsoon	Ferralsol	15.6	1420
Xing et al. (2024)‡	–	119.72	30.38	–	Greenhouse / pot experiment	Ferralsol	–	–
Xu et al. (2020)‡	Banqiao, Zhejiang	119.75	30.17	90–200	Subtropical humid monsoon	Ferralsol	15.9	1350–1500
Xu et al. (2022)	Miaoshanwu Nature Reserve, Fuyang, Zhejiang	119.95	29.48	130	Subtropical monsoon	Ferralsol	16.1	1442
Xu et al. (2024)	Lin'an, Hangzhou, Zhejiang	119.72	30.38	–	Greenhouse / pot experiment	Ferralsol	–	–
Yang et al. (2017)	Qingshan Township, Lin'an, Zhejiang	118.85	29.93	150–200	Subtropical monsoon	Ferralsol	16.0	1450
Yang et al. (2018)	Tianhuangping, Anji, Zhejiang	119.62	30.52	300–380	Subtropical monsoon	Ferric Luvisol	16.0	1300
Yang et al. (2022)‡	Lin'an, Hangzhou, Zhejiang	119.70	30.23	100–300	Subtropical monsoon	Ferralsol	15.6	1420
Yang et al. (2025a)	Maowu, Daixi, Huzhou, Zhejiang	120.00	30.65	65	Northern subtropical monsoon	Red soil	12.2–17.3	1278
Yang et al. (2025b)‡	Research Institute of Subtropical Forestry, Hangzhou, Zhejiang	119.95	29.80	93	Greenhouse / pot experiment	Red soil	–	–
Yang et al. (2025c)	Maowu, Wuxing, Huzhou, Zhejiang	120.00	30.68	75–79	Northern subtropical monsoon	Red soil	12.2–17.3	761–1780

Articles	Location	Long.	Lat.	Alt. (m)	Climate	Soil	Temp (°C)	Pre (mm)
Yang et al. (2025d)‡	Renzhou, Sandiejin National Forest Park, Fujian	119.17	26.23	–	Subtropical maritime monsoon	Red soil	19.6	900–2100
Yuan et al. (2022)‡	–	116.36	39.91	–	Greenhouse / pot experiment	–	–	–
Zeng et al. (2024a)	Changning, Sichuan	105.02	28.45	260–1000	Subtropical humid monsoon	Cambisol	18.3	1142
Zeng et al. (2024b)	Daiyun Mountain, Dehua, Fujian	118.33	25.72	1200	Subtropical humid monsoon	Ultisol	21.2	1800
Zhang et al. (2013)‡	Yuhang County, Zhejiang	120.30	30.42	–	Subtropical monsoon	Ultisol	15.7	1550
Zhang et al. (2014)	Yuhang, Zhejiang	119.92	30.29	–	Subtropical monsoon	Ultisol	15.7	1550
Zhang et al. (2017b)‡	Lin'an, Zhejiang	119.70	30.23	100–300	Subtropical monsoon	Ferralsol	15.6	1420
Zhang et al. (2019a)‡	Guixi	117.35	27.90	800	–	–	17.5	1800
Zhang et al. (2019b)	Guan Shan Forestry Farm	115.48	27.22	–	–	Red soil	16.2	1950–2100
Zhang et al. (2020)	Quanwang, Quzhou, Zhejiang	118.92	28.93	–	Subtropical humid monsoon	Yellow-brown soil	17.3	1667
Zhang et al. (2021a)	Anji, Zhejiang	119.63	30.48	235–238	Subtropical monsoon	Ferralsol	16.1	1423
Zhang et al. (2021b)	Qingshan Town, Zhejiang	119.70	30.23	100–300	Subtropical monsoon	Ferralsol	15.6	1420
Zhang et al. (2021c)	Qingshan, Hangzhou, Zhejiang	118.85	29.93	150–200	Subtropical monsoon	Ferralsol	16.0	1450
Zhang et al. (2023)	Qingshan, Hangzhou, Zhejiang	119.78	30.22	120	Subtropical monsoon	Ferralsol	15.6	1420
Zhao et al. (2016)	Huangshan, Anhui	118.23	32.07	492	Mid-subtropical humid monsoon	Ultisol	15.3	1500
Zhao et al. (2019)‡	Huangshan, Anhui	118.23	32.07	492	Mid-subtropical humid monsoon	Ultisol	15.3	1500
Zhao et al. (2024)	Taihuyuan, Hangzhou, Zhejiang	119.58	30.25	–	Subtropical monsoon	Red soil	16.4	1629
Zheng et al. (2022)	Huzhou, Zhejiang	120.09	30.87	–	–	Siltstone-derived red soil	15.6	1309
Zheng et al. (2024)	Quanzhou, Fujian	118.16	25.66	–	Subtropical monsoon	Ultisol	19.5	1769

Articles	Location	Long.	Lat.	Alt. (m)	Climate	Soil	Temp (°C)	Pre (mm)
Zhou et al. (2021)	Qingshan Township, Hangzhou	118.85	29.93	150–200	Subtropical monsoon	Ferralsol	16.0	1450
Zhou et al. (2024)	Lin'an	119.78	30.22	120	Subtropical monsoon	Ferralsol	15.6	1420
Zhou et al. (2026)	Qingshan Township, Hangzhou, Zhejiang	119.78	30.22	120	Subtropical monsoon	Ferralsol	15.6	1420

Note: Four experiments were each reported in two articles (Cheng et al., 2025a, 2025b; Huang et al., 2025a, 2026; Jiang et al., 2025a, 2026a; Yang et al., 2025a, 2025c); therefore, the 98 articles listed here describe 94 independent studies.

Table S4a. Publication bias: Rosenberg weighted fail-safe numbers for every fertilization regime × variable combination presented in Figures 1–3 that met the three-study threshold, calculated from all paired observations of each combination before outlier screening. An effect is considered robust to publication bias when the fail-safe number exceeds 5n + 10, where n is the number of observations (Rosenberg, 2005). Weights are the inverse sampling variances of Equation (2). Following the convention of METAWIN, a fail-safe number is reported as zero where the formula returns a negative value. Fail-safe numbers are calculated for each regime separately rather than for each variable, because the conclusions of this study are drawn for each fertilization regime; pooling regimes with opposing responses would offset their effects and understate the evidence.

Variable	Regime	n	5n + 10	Fail-safe number
Soil pH	N	35	185	23,867
Soil pH	NPK	83	425	8,020
Soil pH	N+OM	30	160	2,784
Soil pH	B	16	90	20,049
Soil moisture	N	220	1,110	2,056
Soil moisture	NPK	139	705	29,994
Soil moisture	N+OM	296	1,490	10,954
Soil moisture	B	160	810	9,761
Soil organic carbon	N	62	320	384
Soil organic carbon	NPK	121	615	11,922
Soil organic carbon	N+OM	38	200	17,326
Soil organic carbon	B	20	110	1,291
Total nitrogen	N	34	180	2,721
Total nitrogen	NPK	53	275	3,006
Total nitrogen	N+OM	26	140	8,967
Total nitrogen	B	18	100	82
Dissolved organic carbon	N	214	1,080	79
Dissolved organic carbon	NPK	107	545	394
Dissolved organic carbon	N+OM	248	1,250	110,187
Dissolved organic carbon	B	47	245	52,048
Dissolved organic nitrogen	N	75	385	1,632
Dissolved organic nitrogen	NPK	62	320	18,069
Dissolved organic nitrogen	N+OM	84	430	1,567
Ammonium nitrogen (NH_4^+-N)	N	157	795	60,697
Ammonium nitrogen (NH_4^+-N)	NPK	114	580	175,299
Ammonium nitrogen (NH_4^+-N)	N+OM	180	910	34,437
Ammonium nitrogen (NH_4^+-N)	B	16	90	2,140
Nitrate nitrogen (NO_3^--N)	N	153	775	226,037
Nitrate nitrogen (NO_3^--N)	NPK	86	440	83,606
Nitrate nitrogen (NO_3^--N)	N+OM	172	870	54,972
Available phosphorus	N	72	370	112,518
Available phosphorus	NPK	57	295	20,707
Available phosphorus	N+OM	18	100	8,631
Available phosphorus	B	14	80	0
Total phosphorus	N	14	80	0

Total phosphorus	NPK	44	230	0
Total phosphorus	N+OM	20	110	2,070
Total phosphorus	B	14	80	106
Microbial biomass carbon	N	427	2,145	941,997
Microbial biomass carbon	NPK	215	1,085	92,683
Microbial biomass carbon	N+OM	494	2,480	437,223
Microbial biomass carbon	B	96	490	3,591
Microbial biomass nitrogen	N	132	670	274
Microbial biomass nitrogen	NPK	121	615	32,019
Microbial biomass nitrogen	N+OM	163	825	7,865
Microbial biomass nitrogen	B	21	115	23
Urease activity	N	61	315	616
Urease activity	NPK	93	475	596,075
Urease activity	N+OM	86	440	3,099
β-Glucosidase activity	N	91	465	2,463
β-Glucosidase activity	NPK	44	230	11,274
β-Glucosidase activity	N+OM	130	660	7,646
Invertase activity	N	98	500	3,667
Invertase activity	NPK	55	285	2,631
Invertase activity	N+OM	137	695	3,619
Soil CO_2 efflux	N	573	2,875	1,511,860
Soil CO_2 efflux	NPK	79	405	25,888
Soil CO_2 efflux	N+OM	507	2,545	1,396,937
Soil CO_2 efflux	B	232	1,170	45,783
Cumulative soil CO_2 efflux	N	43	225	24,741
Cumulative soil CO_2 efflux	N+OM	19	105	4,052
Cumulative soil CO_2 efflux	B	13	75	25
N_2O emission rate	N	302	1,520	91,503
N_2O emission rate	NPK	51	265	22,428
N_2O emission rate	N+OM	439	2,205	118,525
Cumulative N_2O emission	N	15	85	743
Cumulative N_2O emission	NPK	5	35	5,484
Cumulative N_2O emission	N+OM	13	75	6
Cumulative CH_4 uptake	N	18	100	487
Cumulative CH_4 uptake	N+OM	16	90	155

Table S4b. Egger-type test for funnel asymmetry for each variable. A meta-regression of lnRR on its standard error with fertilization regime as a moderator and a study random intercept, tested against a t distribution with degrees of freedom equal to the number of studies minus the number of model parameters; $P < 0.05$ indicates funnel asymmetry. Degrees of freedom are counted from the number of studies rather than the number of observations, because observations within a study are not independent.

Variable	n	Studies	Egger t	df	Egger P	Interpretation
Soil pH	164	29	+0.96	24	0.346	–
Soil moisture	815	16	+0.10	11	0.919	–
Soil organic carbon	241	32	−0.70	27	0.487	–
Total nitrogen	131	24	−0.04	19	0.966	–
Dissolved organic carbon	616	22	+3.14	17	0.006	Asymmetry
Dissolved organic nitrogen	226	12	−2.15	7	0.069	–
Ammonium nitrogen (NH_4^+-N)	467	24	+0.85	19	0.408	–
Nitrate nitrogen (NO_3^--N)	416	22	+3.47	17	0.003	Asymmetry
Available phosphorus	161	24	−1.25	19	0.226	–
Total phosphorus	92	16	−0.03	11	0.980	–
Microbial biomass carbon	1,232	29	+0.77	24	0.448	–
Microbial biomass nitrogen	437	16	−2.38	11	0.036	Asymmetry
Urease activity	244	11	+0.78	6	0.462	–
β-Glucosidase activity	303	9	−2.74	4	0.052	–
Invertase activity	330	8	−0.64	3	0.569	–
Soil CO_2 efflux	1,391	14	+1.99	9	0.078	–
Cumulative soil CO_2 efflux	79	12	−3.72	7	0.008	Asymmetry
N_2O emission rate	914	7	+5.19	2	0.035	Asymmetry
Cumulative N_2O emission	35	7	+1.70	2	0.231	–
Cumulative CH_4 uptake	43	6	−0.04	1	0.973	–

Table S5. Sensitivity of the pooled effects to the analytical choices. Log response ratio (lnRR, Equation 1) of each fertilization regime relative to the unfertilized control. DerSimonian–Laird, the analysis reported in Figures 1–3; REML, the same observations and the same weights of Equation (3), with the between-study variance estimated by restricted maximum likelihood; No outlier removal, the DerSimonian–Laird estimate with observations whose standardized residuals exceed three retained; Field only, the DerSimonian–Laird estimate with pot experiments and laboratory incubations excluded. n, number of observations entering the main estimate; Removed, observations excluded by the residual screen. * $P < 0.05$, ** $P < 0.01$, *** $P < 0.001$.

		DerSimonian–Laird					
**Variable**	**Regime**	**n**	**Removed**	**Outlier removal**	**No outlier removal**	**REML**	**Field only**
Soil pH	N	34	1	−0.054***	−0.051***	−0.054***	−0.054***
Soil pH	NPK	81	2	−0.017*	−0.020*	−0.017	−0.017*
Soil pH	N+OM	29	1	+0.033**	+0.033**	+0.033*	+0.031**
Soil pH	B	15	1	+0.090***	+0.090***	+0.090***	+0.091***
Soil moisture	N	218	2	+0.060*	+0.059*	+0.060*	+0.031***
Soil moisture	NPK	137	2	−0.040***	−0.036**	−0.040***	−0.040***
Soil moisture	N+OM	290	6	+0.038*	+0.077***	+0.038**	+0.006
Soil moisture	B	159	1	+0.015	+0.192***	+0.015	+0.025***
Soil organic carbon	N	61	1	−0.121***	−0.030	−0.121***	−0.128***
Soil organic carbon	NPK	121	0	+0.165**	+0.165**	+0.165**	+0.165**
Soil organic carbon	N+OM	38	0	+0.252***	+0.252***	+0.252***	+0.235***
Soil organic carbon	B	18	2	+0.131**	+0.141*	+0.131	+0.130***
Total nitrogen	N	34	0	−0.096	−0.096	−0.096	−0.098
Total nitrogen	NPK	50	3	+0.071	+0.068	+0.071	+0.071
Total nitrogen	N+OM	26	0	+0.288***	+0.288***	+0.288***	+0.289***
Total nitrogen	B	18	0	+0.049	+0.049	+0.049	+0.046
Dissolved organic carbon	N	208	6	−0.102***	−0.116**	−0.102***	−0.102***
Dissolved organic carbon	NPK	98	9	−0.010	−0.024	−0.010	−0.010
Dissolved organic carbon	N+OM	243	5	+0.096***	+0.105***	+0.096***	+0.096***
Dissolved organic carbon	B	47	0	+0.450*	+0.450*	+0.450**	+0.450*
Dissolved organic nitrogen	N	71	4	+0.108***	+0.124***	+0.108***	+0.108***
Dissolved organic nitrogen	NPK	60	2	+0.058	+0.052	+0.058	+0.058
Dissolved organic nitrogen	N+OM	83	1	−0.006	−0.001	−0.006	−0.006
Ammonium nitrogen (NH_4^+-N)	N	148	9	+0.122***	+0.148***	+0.122***	+0.122***
Ammonium nitrogen (NH_4^+-N)	NPK	109	5	+0.127	+0.129	+0.127	+0.127
Ammonium nitrogen (NH_4^+-N)	N+OM	168	12	−0.032	−0.017	−0.032	−0.032
Ammonium nitrogen (NH_4^+-N)	B	16	0	−0.184	−0.184	−0.184	−0.184
Nitrate nitrogen (NO_3^--N)	N	153	0	+0.547***	+0.547***	+0.547***	+0.547***
Nitrate nitrogen (NO_3^--N)	NPK	83	3	+0.505***	+0.507***	+0.505***	+0.505***
Nitrate nitrogen (NO_3^--N)	N+OM	168	4	+0.048	+0.069*	+0.048	+0.048
Available phosphorus	N	72	0	+0.411**	+0.411**	+0.411**	+0.516***
Available phosphorus	NPK	57	0	−0.638	−0.638	−0.638	−0.638
Available phosphorus	N+OM	18	0	−0.376	−0.376	−0.376	−0.376
Available phosphorus	B	14	0	+0.073	+0.073	+0.073	+0.073
Total phosphorus	N	14	0	+0.002	+0.002	+0.002	+0.002

Total phosphorus	NPK	44	0	+0.059	+0.059	+0.059	+0.059
Total phosphorus	N+OM	20	0	+0.511***	+0.511***	+0.511**	+0.511***
Total phosphorus	B	14	0	+0.088	+0.088	+0.088	+0.088
Microbial biomass carbon	N	421	6	+0.187***	+0.154***	+0.187***	+0.187***
Microbial biomass carbon	NPK	209	6	+0.101***	+0.089**	+0.101***	+0.101***
Microbial biomass carbon	N+OM	482	12	+0.093***	+0.095***	+0.093***	+0.093***
Microbial biomass carbon	B	94	2	+0.057	+0.055	+0.057	+0.057
Microbial biomass nitrogen	N	132	0	−0.007	−0.007	−0.007	−0.007
Microbial biomass nitrogen	NPK	119	2	+0.071	+0.072	+0.071	+0.071
Microbial biomass nitrogen	N+OM	158	5	+0.134***	+0.127**	+0.134***	+0.134***
Microbial biomass nitrogen	B	21	0	+0.099***	+0.099***	+0.099**	+0.099***
Urease activity	N	60	1	+0.013	−0.011	+0.013	−0.073
Urease activity	NPK	93	0	−0.090	−0.090	−0.090	−0.090
Urease activity	N+OM	84	2	−0.065	−0.059	−0.065	−0.065
β-Glucosidase activity	N	90	1	+0.074*	−0.144*	+0.074*	+0.074*
β-Glucosidase activity	NPK	44	0	+0.077	+0.077	+0.077	+0.077
β-Glucosidase activity	N+OM	129	1	+0.134	+0.134	+0.134	+0.134
Invertase activity	N	97	1	−0.056	−0.055	−0.056	−0.046
Invertase activity	NPK	54	1	+0.109	+0.108	+0.109	+0.109
Invertase activity	N+OM	137	0	−0.133	−0.133	−0.133	−0.135
Soil CO_2 efflux	N	563	10	+0.126***	+0.140***	+0.126***	+0.126***
Soil CO_2 efflux	NPK	78	1	+0.200***	+0.207***	+0.200***	+0.200***
Soil CO_2 efflux	N+OM	501	6	+0.189***	+0.191***	+0.189***	+0.189***
Soil CO_2 efflux	B	227	5	+0.028	+0.032	+0.028	+0.028
Cumulative soil CO_2 efflux	N	43	0	+0.200***	+0.200***	+0.200***	+0.200***
Cumulative soil CO_2 efflux	N+OM	19	0	+0.256***	+0.256***	+0.256***	+0.256***
Cumulative soil CO_2 efflux	B	12	1	+0.131	+0.111	+0.131*	+0.131
N_2O emission rate	N	292	10	+0.139***	+0.156***	+0.139***	+0.139***
N_2O emission rate	NPK	49	2	+0.352***	+0.356***	+0.352***	+0.352***
N_2O emission rate	N+OM	422	17	−0.061*	−0.059	−0.061	−0.061*
Cumulative N_2O emission	N	14	1	+0.172***	+0.180***	+0.172***	+0.172***
Cumulative N_2O emission	NPK	5	0	+0.600***	+0.600***	+0.600***	+0.600***
Cumulative N_2O emission	N+OM	13	0	−0.010	−0.010	−0.010	−0.010
Cumulative CH_4 uptake	N	18	0	−0.169***	−0.169***	−0.169***	−0.169***
Cumulative CH_4 uptake	N+OM	16	0	+0.086***	+0.086***	+0.086***	+0.086***

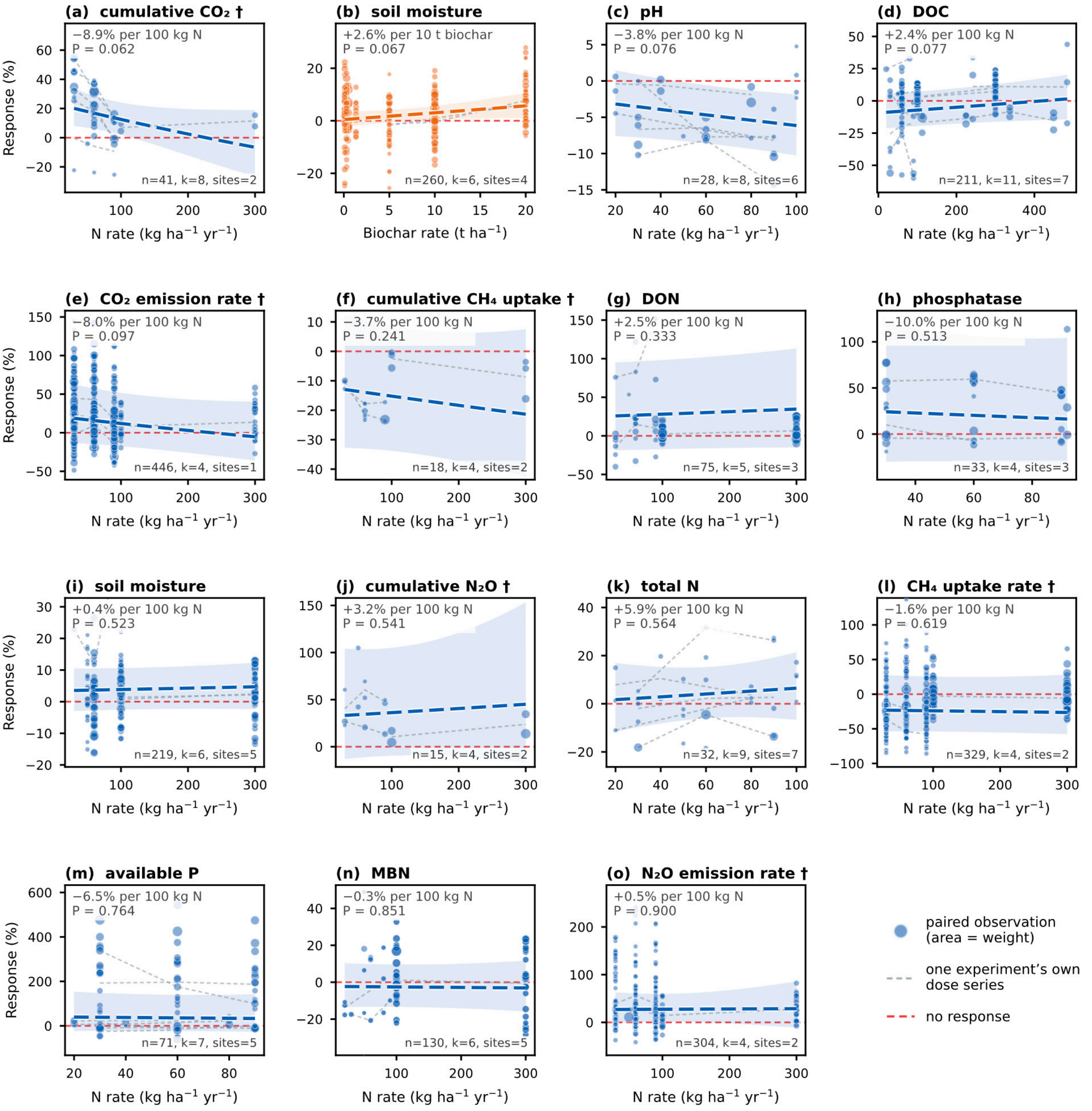

Figure S1. Response of the remaining soil, microbial, and greenhouse gas variables to fertilizer application rate. Panel (b) is on the biochar application rate axis and all others on the nitrogen application rate axis. Circles are paired observations scaled by their weight, blue for nitrogen and orange for biochar, and gray dashed lines join the conditions of a single experiment. The dashed line is the fitted meta-regression of *lnRR* on application rate and the shaded band its 95% confidence interval; none of these slopes differs from zero at $P < 0.05$. The red dashed line marks no change. Each panel gives the change per 100 kg N ha^{-1} yr^{-1} or per 10 t biochar ha^{-1}, the P value, and the number of paired observations (n), studies (k) and sites. The dagger marks panels based on fewer than three sites.

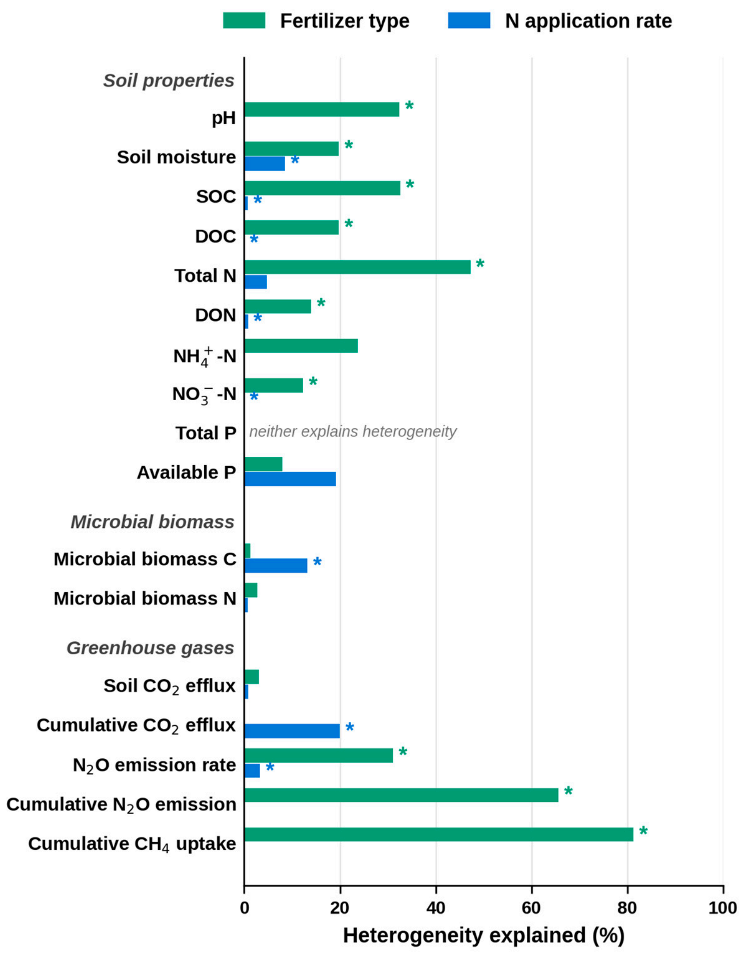

Figure S2. Share of the between-study heterogeneity explained by the type of input (fertilization regime) and by the nitrogen application rate. Both moderators were fitted to the same observations and weights from the nitrogen-bearing regimes (N, NPK and N+OM; N and N+OM for cumulative soil CO_2 efflux and cumulative CH_4 uptake, and N and NPK for TP), and the share was calculated as $(\tau^2_{null} - \tau^2_{moderator})/\tau^2_{null}$. Asterisks mark moderators with $P < 0.05$. Neither moderator explained any heterogeneity for TP.

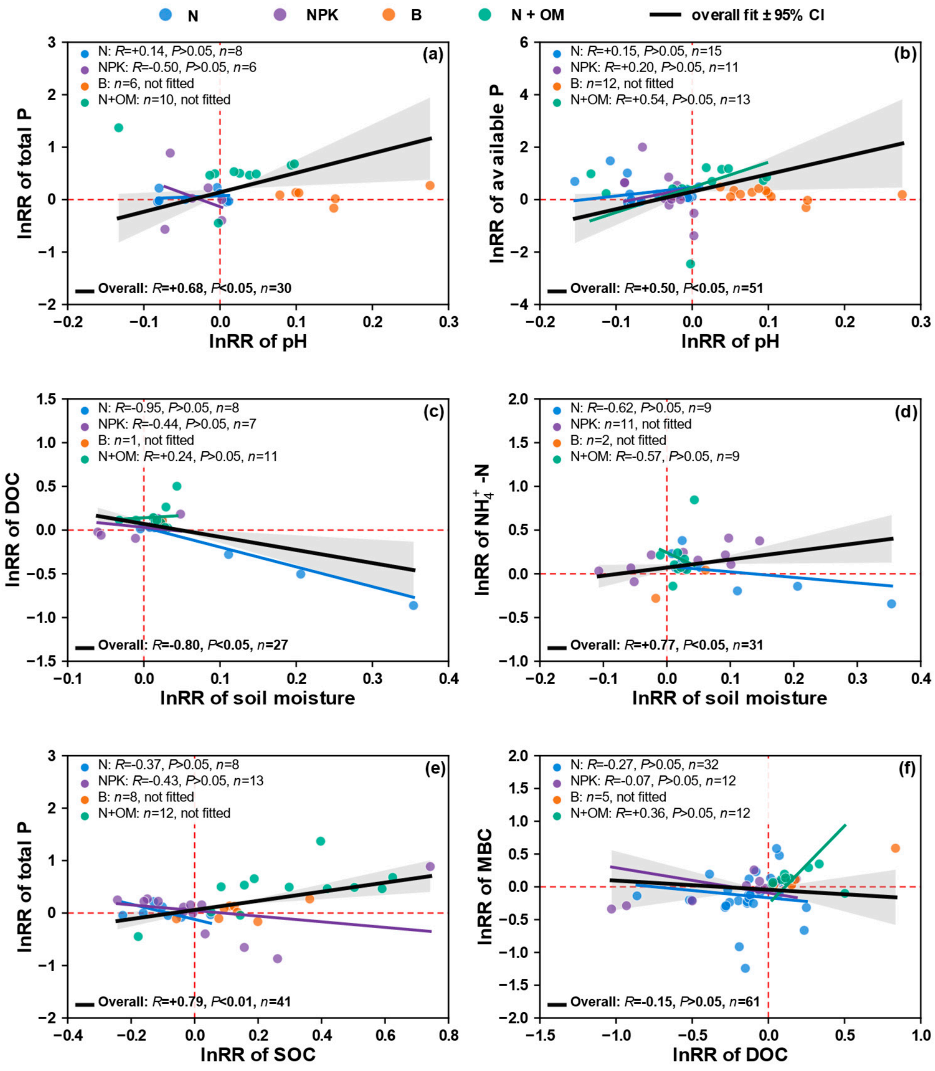

Figure S3. Covariation among the additional soil and nutrient responses across matched treatment conditions. (a) TP against pH, (b) AP against pH, (c) DOC against SM, (d) NH_4^+-N against SM, (e) TP against SOC and (f) MBC against DOC. Axes are log response ratios, and the red dashed lines mark zero on both axes. Each point is one matched condition, colored by fertilization regime, and n is the number of matched conditions. Colored lines are the regime-specific slopes from a single model in which the four regimes share one variance estimate; a regime is fitted only where it supplied at least six matched conditions from at least four independent studies, and is otherwise shown as points alone and marked "not fitted". The black line is the overall fit and the shaded band its 95% confidence interval. Slopes are estimated within studies with a study-level random effect, and R is the corresponding partial correlation.

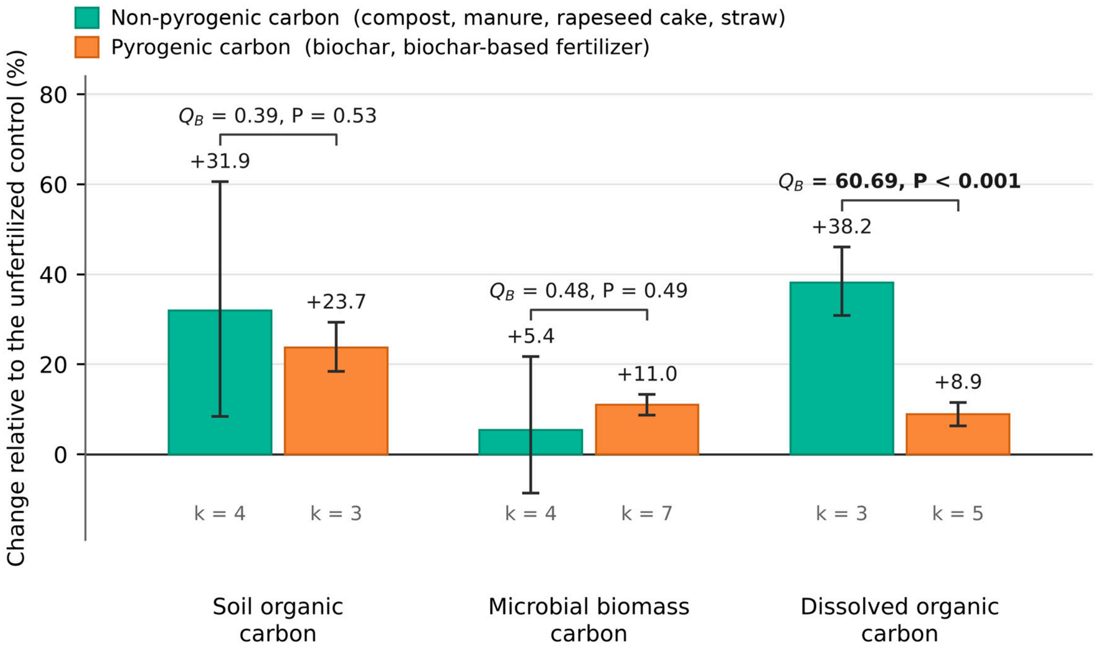

Figure S4. Response of the soil carbon pools to nitrogen applied with organic matter, separated by the form of the carbon added. Non-pyrogenic carbon comprises compost, manure, rapeseed cake and straw; pyrogenic carbon comprises biochar and biochar-based fertilizers. Bars are the mean percent change relative to the unfertilized control with 95% confidence intervals; k is the number of contributing studies. Q_B tests the difference between the two forms. No difference between carbon forms was detected for soil organic carbon or microbial biomass carbon, whereas dissolved organic carbon increased more under non-pyrogenic carbon, consistent with its greater lability.

Data Source

Cao, L., Yu, X., Liu, C., Liu, M., Chen, J., Qin, H., et al. (2020) Alteration of soil nitrifiers and denitrifiers and their driving factors during intensive management of Moso bamboo (Phyllostachys pubescens). Science of The Total Environment, 705, 135236. https://doi.org/10.1016/j.scitotenv.2019.135236.

Chen, J., Wu, Q., Li, S., Ge, J., Liang, C., Qin, H., et al. (2019) Diversity and function of soil bacterial communities in response to long-term intensive management in a subtropical bamboo forest. Geoderma, 354, 113894. https://doi.org/10.1016/j.geoderma.2019.113894.

Chen, H., Cheng, X., Zhang, X., Shi, H., Chen, J., Xu, R., et al. (2024) Effects of Fertilizer Application Intensity on Carbon Accumulation and Greenhouse Gas Emissions in Moso Bamboo Forest–Polygonatum cyrtonema Hua Agroforestry Systems. Plants, 13, 1941. https://doi.org/10.3390/plants13141941.

Chen, L., Zeng, Q., Zhang, Q., Zhu, B., Fan, Y., Yuan, X. and Chen, Y. (2025) Microbial phosphorus demand affects carbon-degrading potential under long-term nitrogen addition in a subtropical forest. Applied Soil Ecology, 211, 106154. https://doi.org/10.1016/j.apsoil.2025.106154.

Chen, Y., Liu, Q., Chen, X., Chen, J., Zhu, B., Fu, S., Chang, S. X. and Cai, Y. (2026a) Canopy and understory nitrogen additions differentially regulate soil organic carbon fractions via litter-microbe-mineral interactions. Forest Ecosystems, 15, 100410. https://doi.org/10.1016/j.fecs.2025.100410.

Chen, Z., Chen, D., Qiu, W., Hu, L., Liu, X., Zhu, Q. and Jin, A. (2026b) Effects of biochar-based and conventional sheep manure organic fertilizers on soil properties and microbial communities in a Moso bamboo (Phyllostachys edulis) forest in China. Forests, 17, 659. https://doi.org/10.3390/f17060659.

Cheng, X., Zhou, Y., Zhou, G. and Shi, Y. (2025a) Biochar-based compound fertilizers enhances carbon sequestration and mitigates greenhouse gas emissions in Moso bamboo forests. GCB Bioenergy, 17, e70056. https://doi.org/10.1111/gcbb.70056.

Cheng, X., Wang, G., Zhou, Y., Pan, C., Wang, Z., Zhou, G. and Shi, Y. (2025b) Biofertilizer outcompete chemical fertilizer in enhancing carbon sequestration in Moso bamboo (Phyllostachys edulis (Carriere) J. Houzeau) forests. Industrial Crops and Products, 232, 121244. https://doi.org/10.1016/j.indcrop.2025.121244.

Chu, H., Su, W., Fan, S., He, X. and Huang, Z. (2024a) Impact of Nitrogen Fertilizer Application on Soil Organic Carbon and Its Active Fractions in Moso Bamboo Forests. Forests, 15, 1483. https://doi.org/10.3390/f15091483.

Chu, H., Su, W., Zhou, Y., Wang, Z., Long, Y., Sun, Y., et al. (2024b) Enzyme Activity Stoichiometry Suggests That Fertilization, Especially Nitrogen Fertilization, Alleviates Nutrient Limitation of Soil Microorganisms in Moso Bamboo Forests. Forests, 15, 1040. https://doi.org/10.3390/f15061040.

Deng, X., Yin, J., Xu, L., Shi, Y., Zhou, G., Li, Y., et al. (2020) Effects of abandonment management on soil C and N pools in Moso bamboo forests. Science of The Total Environment, 729, 138949. https://doi.org/10.1016/j.scitotenv.2020.138949.

Gao, P., Zuo, Z., Zhang, R., Qiu, Y., He, R., Gao, R., et al. (2016) Optimum nitrogen fertilization for phyllostachys edulis productivity and photosynthetic response. Agronomy Journal, 108, 448–458. https://doi.org/10.2134/agronj2015.0324.

Gao, G., Xia, J., Xu, Y., Ge, X., Li, A., Zhao, Z., Li, Z. and Zhou, B. (2026) Nitrogen addition alleviates negative effects of drought on root growth but aggravates them on leaf growth of Moso bamboo (Phyllostachys edulis) seedlings. Forest Ecosystems, 15, 100388. https://doi.org/10.1016/j.fecs.2025.100388.

Ge, X., Cao, Y., Zhou, B., Wang, X., Yang, Z. and Li, M.-H. (2019) Biochar addition increases subsurface soil microbial biomass but has limited effects on soil CO_2 emissions in subtropical moso bamboo plantations. Applied Soil Ecology, 142, 155–165. https://doi.org/10.1016/j.apsoil.2019.04.021.

Ge, X., Cao, Y., Zhou, B., Xiao, W., Tian, X. and Li, M.-H. (2020) Combined application of biochar and N increased temperature sensitivity of soil respiration but still decreased the soil CO_2 emissions in moso bamboo plantations. Science of The Total Environment, 730, 139003. https://doi.org/10.1016/j.scitotenv.2020.139003.

He, Y., Tang, Y., Lin, L., Shi, W. and Ying, Y. (2023) Differential responses of phosphorus accumulation and mobilization in Moso bamboo (Phyllostachys edulis (Carrière) J. Houz) seedlings to short-term experimental nitrogen deposition. Annals of Forest Science, 80, 10. https://doi.org/10.1186/s13595-023-01176-w.

He, Y., Luo, C., Zhao, D., Shi, W., Jiang, L., Wu, X., Liu, J., Cao, X., Wang, S., Zhang, M. and Ying, Y. (2026) Does nitrogen and phosphorus addition alter water use traits of Moso bamboo (Phyllostachys edulis (Carriere) J. Houz.) forest in the fast-growing period in subtropical China?. Agricultural Water Management, 331, 110459. https://doi.org/10.1016/j.agwat.2026.110459.

Huang, C., Li, Y., Wu, J., Huang, Z., Chang, S. X. and Jiang, P. (2019) Intensive Management Increases Phytolith-Occluded Carbon Sequestration in Moso Bamboo Plantations in Subtropical China. Forests, 10, 883. https://doi.org/10.3390/f10100883.

Huang, C., Wang, L., Gong, X., Huang, Z., Zhou, M., Li, J., et al. (2020) Silicon fertilizer and biochar effects on plant and soil PhytOC concentration and soil PhytOC stability and fractionation in subtropical bamboo plantations. Science of The Total Environment, 715, 136846. https://doi.org/10.1016/j.scitotenv.2020.136846.

Huang, K., Li, Y., Hu, J., Tang, C., Zhang, S., Fu, S., et al. (2021) Rates of soil respiration components in response to inorganic and organic fertilizers in an intensively-managed Moso bamboo forest. Geoderma, 403, 115212. https://doi.org/10.1016/j.geoderma.2021.115212.

Huang, Z., Li, Q., Bian, F., Zhong, Z. and Zhang, X. (2025a) Effects of bamboo-sourced organic fertilizer on the soil microbial necromass carbon and its contribution to soil organic carbon in Moso bamboo (Phyllostachys edulis) forest. Forests, 16, 553. https://doi.org/10.3390/f16030553.

Huang, Z., Liu, X., Chu, H., Jia, H., He, X., Wang, C., Zhang, B., Pan, C., Liu, S., Fan, S. and Su, W. (2025b) The impact of nitrogen addition on soil carbon components and understory vegetation in Moso bamboo forests. Plants, 14, 569. https://doi.org/10.3390/plants14040569.

Huang, Z., Li, Q., Bian, F., Zhong, Z. and Zhang, X. (2026) Bamboo-sourced organic fertilizer regulates soil microbial nutrient limitation in Moso bamboo plantations: insights from ecological enzyme stoichiometry. Applied Soil Ecology, 224, 107174. https://doi.org/10.1016/j.apsoil.2026.107174.

Jiang, W., Wang, Y., Lin, Y., Akiyama, H., Fang, Y., Vancov, T., Fu, S., Kang, H., Chen, X., Xiong, Z., Li, Z., Hu, S., Li, Y., Yu, B., Chang, S. X. and Cai, Y. (2025a) Both biotic and abiotic soil N_2O productions are lower under organic N than inorganic N deposition in a Moso bamboo forest. Biology and Fertility of Soils, 61, 1271-1285. https://doi.org/10.1007/s00374-025-01934-0.

Jiang, Z., Vancov, T., Fang, Y., Tang, C., Zhang, W., Xiao, M., Song, X., Zhou, J., Ge, T., Cai, Y., Yu, B., White, J. C. and Li, Y. (2025b) Sustained superiority of biochar over straw for enhancing soil biological-phosphorus via the mediation of phoD-harboring bacteria in subtropical Moso bamboo forests. Forest Ecology and Management, 584, 122606. https://doi.org/10.1016/j.foreco.2025.122606.

Jiang, Z., Zhang, S., Tang, C., Xiao, M., Zhou, J., Luo, Y., Ge, T., Yu, B., White, J. C. and Li, Y. (2025c) Divergent effects of straw and biochar on soil carbon priming are depth-dependent in subtropical Moso bamboo forests. Biology and Fertility of Soils, 62, 349-365. https://doi.org/10.1007/s00374-025-01927-z.

Jiang, W., Akiyama, H., Wang, Y., Lin, Y., Zhang, B., Liu, S., Chen, X., Li, Y., Yu, B., Fu, S., Chen, Y., Chang, S. X. and Cai, Y. (2026a) Understory N deposition amplifies the contrasting effects of organic and inorganic N on soil N_2O emissions in a Moso bamboo forest. Industrial Crops and Products, 249, 123624. https://doi.org/10.1016/j.indcrop.2026.123624.

Jiang, Z., Tang, C., Liu, S., Fang, Y., Zhou, J., Xiao, M., Luo, Y., Ge, T., Cai, Y., Yu, B., White, J. C. and Li, Y. (2026b) Nano-zeolite-coupled biochar-based phosphate fertilizer suppresses the priming effect and promotes soil carbon stability in subtropical bamboo plantations. Industrial Crops and Products, 240, 122720. https://doi.org/10.1016/j.indcrop.2026.122720.

Jiang, Z., Tang, C., Fang, Y., Ge, T., Liu, S., Luo, Y., Yu, B., Cai, Y., White, J. C. and Li, Y. (2026c) Nanozeolite-coupled biochar-based phosphate fertilizer dampens warming-induced soil carbon loss by microbial functional constraints in Moso bamboo forests. Biochar, 8, 112. https://doi.org/10.1007/s42773-026-00620-0.

Jin, A., Jin, X., Song, Y., Wang, H. and Zheng, B. (2011) Effect of Fertilization on Photosynthetic Features in Phyllostachys pubescens. The Botanical Review, 77, 304–312. https://doi.org/10.1007/s12229-011-9072-1.

Kim, C., Baek, G., Yoo, B. O., Jung, S.-Y. and Lee, K. S. (2018) Regular Fertilization Effects on the Nutrient Distribution of Bamboo Components in a Moso Bamboo (Phyllostachys pubescens (Mazel) Ohwi) Stand in South Korea. Forests, 9, 671. https://doi.org/10.3390/f9110671.

Lei, Z., Sun, H., Li, Q., Zhang, J. and Song, X. (2017) Effects of Nitrogen Deposition on Soil Dissolved Organic Carbon and Nitrogen in Moso Bamboo Plantations Strongly Depend on Management Practices. Forests, 8, 452. https://doi.org/10.3390/f8110452.

Li, Y., Zhang, J., Chang, S. X., Jiang, P., Zhou, G., Fu, S., et al. (2013) Long-term intensive management effects on soil organic carbon pools and chemical composition in Moso bamboo (Phyllostachys pubescens) forests in subtropical China. Forest Ecology and Management, 303, 121–130. https://doi.org/10.1016/j.foreco.2013.04.021.

Li, Q., Song, X., Gu, H. and Gao, F. (2016) Nitrogen deposition and management practices increase soil microbial biomass carbon but decrease diversity in Moso bamboo plantations. Scientific Reports, 6, 28235. https://doi.org/10.1038/srep28235.

Li, C., Shi, Y., Zhou, G., Zhou, Y., Xu, L., Tong, L., et al. (2018a) Effects of different management approaches on soil carbon dynamics in Moso bamboo forest ecosystems. CATENA, 169, 59–68. https://doi.org/10.1016/j.catena.2018.05.031.

Li, Q., Lei, Z., Song, X., Zhang, Z., Ying, Y. and Peng, C. (2018b) Biochar amendment decreases soil microbial biomass and increases bacterial diversity in Moso bamboo (Phyllostachys edulis) plantations under simulated nitrogen deposition. Environmental Research Letters, 13, 044029. https://doi.org/10.1088/1748-9326/aab53a.

Li, Q., Song, X., Chang, S. X., Peng, C., Xiao, W., Zhang, J., et al. (2019) Nitrogen depositions increase soil respiration and decrease temperature sensitivity in a Moso bamboo forest. Agricultural and Forest Meteorology, 268, 48–54. https://doi.org/10.1016/j.agrformet.2019.01.012.

Li, W., Sheng, H., Liu, Y., Zhang, R., Ekawati, D., Qian, Y., et al. (2020a) Ecostoichiometry Reveals the Separation of Microbial Adaptation Strategies in a Bamboo Forest in an Urban Wetland under Simulated Nitrogen Deposition. Forests, 11, 428. https://doi.org/10.3390/f11040428.

Li, W., Tian, X., Sheng, H., Ekawati, D., Zhou, Y. and Zhang, R. (2020b) Response of bacterial compositions to soil biochemical properties under mulching-intensive management in a Phyllostachys edulis forest. Applied Soil Ecology, 150, 103436. https://doi.org/10.1016/j.apsoil.2019.103436.

Li, Q., Lv, J., Peng, C., Xiang, W., Xiao, W. and Song, X. (2021a) Nitrogen -addition accelerates phosphorus cycling and changes phosphorus use strategy in a subtropical Moso bamboo forest. Environmental Research Letters, 16, 024023. https://doi.org/10.1088/1748-9326/abd5e1.

Li, Q., Peng, C., Zhang, J., Li, Y. and Song, X. (2021b) Nitrogen addition decreases methane uptake caused by methanotroph and methanogen imbalances in a Moso bamboo forest. Scientific Reports, 11, 5578. https://doi.org/10.1038/s41598-021-84422-3.

Li, Q., Cui, K., Lv, J., Zhang, J., Peng, C., Li, Y., et al. (2022a) Biochar amendments increase soil organic carbon storage and decrease global warming potentials of soil CH_4 and N_2O under N addition in a subtropical Moso bamboo plantation. Forest Ecosystems, 9, 100054. https://doi.org/10.1016/j.fecs.2022.100054.

Li, Q., Ma, Q., Gao, J., Zhang, J., Li, Y., Shi, M., et al. (2022b) Stumps increased soil respiration in a subtropical Moso bamboo (Phyllostachys edulis) plantation under nitrogen addition. Agricultural and Forest Meteorology, 323, 109047. https://doi.org/10.1016/j.agrformet.2022.109047.

Li, Q., Zhang, C., Shi, M., Lv, J., Peng, C., Zhang, J., et al. (2024a) Long-term nitrogen addition has a positive legacy effect on soil respiration in subtropical Moso bamboo forests. Geoderma, 452, 117092. https://doi.org/10.1016/j.geoderma.2024.117092.

Li, Y., Zhang, S., Fang, Y., Hui, D., Tang, C., Van Zwieten, L., et al. (2024b) Nitrogen deposition-induced stimulation of soil heterotrophic respiration is counteracted by biochar in a subtropical forest. Agricultural and Forest Meteorology, 349, 109940. https://doi.org/10.1016/j.agrformet.2024.109940.

Li, Z., Guan, F., Zhou, X., Liu, L., Fu, D., Zhang, X., et al. (2024c) Effect of Fertilization on Soil Fertility and Individual Stand Biomass in Strip Cut Moso Bamboo (Phyllostachys edulis) Forests. Forests, 15, 252. https://doi.org/10.3390/f15020252.

Li, Q., Huang, Z., Bian, F., Zhong, Z. and Zhang, X. (2025) Bamboo-sourced microbial fertilizers impact soil carbon cycling: metagenomic insights from Moso bamboo plantations. Environmental Technology & Innovation, 40, 104548. https://doi.org/10.1016/j.eti.2025.104548.

Liu, J., Jiang, P., Li, Y., Zhou, G., Wu, J. and Yang, F. (2011) Responses of N_2O Flux from Forest Soils to Land Use Change in Subtropical China. The Botanical Review, 77, 320–325. https://doi.org/10.1007/s12229-011-9074-z.

Liu, C., Shi, Y., Chen, J., Qin, H., Liang, C., Fuhrmann, J. J., et al. (2023a) Distinct response of soil CO_2- and N2-fixing bacteria to long-term application of mineral fertilizer combined with manure in a subtropical bamboo forest. Journal of Soils and Sediments, 23, 390–404. https://doi.org/10.1007/s11368-022-03301-0.

Liu, L., Chang, S. X., Huang, C., Zhi, Y., Jie, Y., Yu, X., et al. (2023b) Enhancement of phytolith-occluded carbon accumulation of Moso bamboo response to temperatures elevation and different fertilization. Frontiers in Plant Science, Volume 14 - 2023. https://doi.org/10.3389/fpls.2023.1144961.

Liu, X., Tang, X., Ran, H., Deng, M., Song, Q., Yang, Q., et al. (2023c) Si Supply Could Alter N Uptake and Assimilation of Saplings—A 15N Tracer Study of Four Subtropical Species. Forests, 14, 1353. https://doi.org/10.3390/f14071353.

Luan, J., Li, S., Wang, Y., Ding, L., Cai, C. and Liu, S. (2022) Decomposition of diverse litter mixtures affected by drought depends on nitrogen and soil fauna in a bamboo forest. Soil Biology and Biochemistry, 173, 108783. https://doi.org/10.1016/j.soilbio.2022.108783.

Lv, W., Zhou, G., Chen, G., Zhou, Y., Ge, Z., Niu, Z., et al. (2020) Effects of Different Management Practices on the Increase in Phytolith-Occluded Carbon in Moso Bamboo Forests. Frontiers in Plant Science, Volume 11 - 2020. https://doi.org/10.3389/fpls.2020.591852.

Lv, J., Li, Q., Zhang, J., Li, Y., Peng, C. and Song, X. (2022) Nitrogen addition increases CO_2, CH_4, and N_2O concentrations of topsoils and subsoils in a subtropical Moso bamboo forest. CATENA, 216, 106397. https://doi.org/10.1016/j.catena.2022.106397.

Ni, H., Su, W., Fan, S. and Chu, H. (2021) Effects of intensive management practices on rhizosphere soil properties, root growth, and nutrient uptake in Moso bamboo plantations in subtropical China. Forest Ecology and Management, 493, 119083. https://doi.org/10.1016/j.foreco.2021.119083.

Peng, C., Li, Q., Zhang, Z., Wu, Z., Song, X., Zhou, G., et al. (2019) Biochar amendment changes the effects of nitrogen deposition on soil enzyme activities in a Moso bamboo plantation. Journal of Forest Research, 24, 275–284. https://doi.org/10.1080/13416979.2019.1646970.

Qin, H., Chen, J., Wu, Q., Niu, L., Li, Y., Liang, C., et al. (2017) Intensive management decreases soil aggregation and changes the abundance and community compositions of arbuscular mycorrhizal fungi in Moso bamboo (Phyllostachys pubescens) forests. Forest Ecology and Management, 400, 246–255. https://doi.org/10.1016/j.foreco.2017.06.003.

Song, X., Zhou, G., Gu, H. and Qi, L. (2015) Management practices amplify the effects of N deposition on leaf litter decomposition of the Moso bamboo forest. Plant and Soil, 395, 391–400. https://doi.org/10.1007/s11104-015-2578-2.

Song, X., Peng, C., Ciais, P., Li, Q., Xiang, W., Xiao, W., et al. (2020) Nitrogen addition increased CO₂ uptake more than non-CO₂ greenhouse gases emissions in a Moso bamboo forest. Science Advances, 6, eaaw5790. https://doi.org/10.1126/sciadv.aaw5790.

Su, W., Fan, S., Zhao, J. and Cai, C. (2019) Effects of various fertilization placements on the fate of urea-15N in moso bamboo forests. Forest Ecology and Management, 453, 117632. https://doi.org/10.1016/j.foreco.2019.117632.

Su, S., Xiong, X., Han, Y., Di, Y., Jing, Y. and Wang, Y. (2026) Throughfall reduction other than nitrogen addition threatens soil multifunctionality in a subtropical Moso bamboo forest. Microorganisms, 14, 1619. https://doi.org/10.3390/microorganisms14081619.

Sun, X., Liu, S., Tang, C., Zhou, J., Jiang, Z., Dong, C., Luo, Y., Ge, T., White, J. C., Cai, Y., Yu, B. and Li, Y. (2025) Nanozeolite-coupled biochar-based phosphorus fertilizer decreases soil N_2O emissions in a subtropical Moso bamboo forest. Journal of Environmental Management, 394, 127430. https://doi.org/10.1016/j.jenvman.2025.127430.

Wang, Y., Liu, S., Luan, J., Chen, C., Cai, C., Zhou, F., et al. (2021) Nitrogen Addition Exacerbates the Negative Effect of Throughfall Reduction on Soil Respiration in a Bamboo Forest. Forests, 12, 724. https://doi.org/10.3390/f12060724.

Wang, S., Wan, F., Yang, S., Jin, Y., Liang, X., Shi, W., Wang, Q. and Zhao, X. (2026a) Effects of nitrogen-phosphorus fertilization on soil microbial respiration and its temperature sensitivity across soil depths in a Moso bamboo forest. Plant and Soil, 525, 851-863. https://doi.org/10.1007/s11104-026-08805-z.

Wang, X., Wang, N., Zhu, X., Lu, D., Zhou, W., Bai, S. and Chen, H. (2026b) Five-year decomposition of coarse woody debris in a subtropical forest: effects of tree species and nitrogen addition. Forest Ecology and Management, 607, 123588. https://doi.org/10.1016/j.foreco.2026.123588.

Xie, J., Liu, J., Jiang, Z., Xiao, M., Zhou, J., Yu, B. and Li, Y. (2026) Nano-zeolite-coupled biochar-based phosphorus fertilizer enhances soil phosphorus availability and leaf phosphorus concentration in Moso bamboo forests. Frontiers in Plant Science, 17, 1873995. https://doi.org/10.3389/fpls.2026.1873995.

Xing, Y., Wang, F., Yu, S., Zhu, Y., Ying, Y. and Shi, W. (2024) Enhancing Phyllostachys edulis seedling growth in phosphorus-deficient soil: complementing the role of phosphate-solubilizing microorganisms with arbuscular mycorrhizal fungi. Plant and Soil, 497, 449–466. https://doi.org/10.1007/s11104-023-06406-8.

Xu, L., Deng, X., Ying, J., Zhou, G. and Shi, Y. (2020) Silicate fertilizer application reduces soil greenhouse gas emissions in a Moso bamboo forest. Science of The Total Environment, 747, 141380. https://doi.org/10.1016/j.scitotenv.2020.141380.

Xu, Y., Yang, N., Ge, X. and Zhou, B. (2022) Biochar Combined with Nitrogen Alters Rhizosphere Soil Nutrients and Microbial Communities, and Promotes Growth of Moso Bamboo Seedlings. Forests, 13, 1043. https://doi.org/10.3390/f13071043.

Xu, Z., Adusei-Fosu, K., Wang, H., Wang, G. and Dong, D. (2024) Interactive Effects of Biochar and Phosphorus Fertilizer on PhoD-Harboring Bacteria and Phosphorus Dynamics in Moso Bamboo Forest Soil. Journal of Soil Science and Plant Nutrition, 24, 7010–7023. https://doi.org/10.1007/s42729-024-02020-4.

Yang, M., Li, Y., Li, Y., Chang, S. X., Yue, T., Fu, W., et al. (2017) Effects of Inorganic and Organic Fertilizers on Soil CO_2 Efflux and Labile Organic Carbon Pools in an Intensively Managed Moso Bamboo (Phyllostachys pubescens) Plantation in Subtropical China. Communications in Soil Science and Plant Analysis, 48, 332–344. https://doi.org/10.1080/00103624.2016.1269802.

Yang, C., Zhong, Z., Zhang, X., Bian, F. and Du, X. (2018) Responses of Soil Organic Carbon Sequestration Potential and Bacterial Community Structure in Moso Bamboo Plantations to Different Management Strategies in Subtropical China. Forests, 9, 657. https://doi.org/10.3390/f9100657.

Yang, K., Zhu, C., Zhang, J., Li, Z., Liu, Y., Song, X., et al. (2022) Nitrogen fertilization in bamboo forest accelerates the shoot growth and alters the lignification process in shoots. Industrial Crops and Products, 187, 115368. https://doi.org/10.1016/j.indcrop.2022.115368.

Yang, Z., Zhao, J. and Ni, H. (2025a) Silkworm excrement organic fertilizer substitution compound fertilizer improves bamboo shoot yield by altering soil properties and bacterial communities of Moso bamboo (Phyllostachys edulis) forests in subtropical China. Frontiers in Plant Science, 16, 1550946. https://doi.org/10.3389/fpls.2025.1550946.

Yang, Z. and Zhou, B. (2025b) Effects of simulated nitrogen deposition on the physiological and growth characteristics of seedlings of two typical subtropical tree species. Plants, 14, 2153. https://doi.org/10.3390/plants14142153.

Yang, Z.-Y., Zhao, J.-C., Li, Y., Wang, B. and Ni, H.-J. (2025c) Effects of compound fertilizer decrement combined with silkworm excrement organic fertilizer on soil enzyme activities and microbial nutrient limitation in Phyllostachys edulis forests. Chinese Journal of Applied Ecology, 36, 3069-3077. https://doi.org/10.13287/j.1001-9332.202510.006.

Yang, J., Wang, K., Chen, J., Fan, L., Jiang, P. and Zheng, R. (2025d) Long-term fertilizer-based management alters soil N_2O emissions and silicon availability in Moso bamboo forests. Agronomy, 15, 1647. https://doi.org/10.3390/agronomy15071647.

Yuan, T., Zhu, C., Li, G., Liu, Y., Yang, K., Li, Z., et al. (2022) An Integrated Regulatory Network of mRNAs, microRNAs, and lncRNAs Involved in Nitrogen Metabolism of Moso Bamboo. Frontiers in Genetics, Volume 13 - 2022. https://doi.org/10.3389/fgene.2022.854346.

Zeng, C., He, S., Long, B., Zhou, Z., Hong, J., Cao, H., et al. (2024a) Minor Effects of Canopy and Understory Nitrogen Addition on Soil Organic Carbon Turnover Time in Moso Bamboo Forests. Forests, 15, 1144. https://doi.org/10.3390/f15071144.

Zeng, Q., Peñuelas, J., Sardans, J., Zhang, Q., Zhou, J., Yue, K., et al. (2024b) Keystone bacterial functional module activates P-mineralizing genes to enhance enzymatic hydrolysis of organic P in a subtropical forest soil with 5-year N addition. Soil Biology and Biochemistry, 192, 109383. https://doi.org/10.1016/j.soilbio.2024.109383.

Zhang, Q., Shamsi, I. H., Wang, J., Song, Q., Xue, Q., Yu, Y., et al. (2013) Surface runoff and nitrogen (N) loss in a bamboo (Phyllostachys pubescens) forest under different fertilization regimes. Environmental Science and Pollution Research, 20, 4681–4688. https://doi.org/10.1007/s11356-012-1429-4.

Zhang, Q., Wang, J., Wang, G., Shamsi, I. H. and Wang, X. (2014) Site-specific nutrient management in a bamboo forest in Southeast China. Agriculture, Ecosystems & Environment, 197, 264–270. https://doi.org/10.1016/j.agee.2014.08.013.

Zhang, R., Wu, J., Li, Q., Hänninen, H., Peng, C., Yao, H., et al. (2017b) Nitrogen Deposition Enhances Photosynthesis in Moso Bamboo but Increases Susceptibility to Other Stress Factors. Frontiers in Plant Science, Volume 8 - 2017. https://doi.org/10.3389/fpls.2017.01975.

Zhang, M., Zhang, W., Bai, S. H., Niu, Y., Hu, D., Ji, H., et al. (2019a) Minor increases in Phyllostachys edulis (Moso bamboo) biomass despite evident alterations of soil bacterial community structure after phosphorus fertilization alone: Based on field studies at different altitudes. Forest Ecology and Management, 451, 117561. https://doi.org/10.1016/j.foreco.2019.117561.

Zhang, W., Liu, S., Zhang, M., Li, Y., Sheng, K. and Xu, Z. (2019b) Phyllostachys edulis (moso bamboo) rhizosphere increasing soil microbial activity rather than biomass. Journal of Soils and Sediments, 19, 2913–2926. https://doi.org/10.1007/s11368-019-02334-2.

Zhang, X., Gao, G., Wu, Z., Wen, X., Bian, F. and Yang, C. (2020) Biochar-based organic fertilizer application rates for Tetrastigma hemsleyanum planted under Moso bamboo. Journal of Forestry Research, 31, 1813–1821. https://doi.org/10.1007/s11676-019-00965-2.

Zhang, F., Jin, Q., Peng, H. and Zhu, T. (2021a) Soil acidification in moso bamboo (Phyllostachys edulis) forests and changes of soil metal ions (Cu, Pb) concentration. Archives of Agronomy and Soil Science, 67, 1799–1808. https://doi.org/10.1080/03650340.2020.1811237.

Zhang, J., Li, Q., Lv, J., Peng, C., Gu, Z., Qi, L., et al. (2021b) Management scheme influence and nitrogen addition effects on soil CO_2, CH_4, and N_2O fluxes in a Moso bamboo plantation. Forest Ecosystems, 8, 6. https://doi.org/10.1186/s40663-021-00285-0.

Zhang, S., Li, Y., Singh, B. P., Wang, H., Cai, X., Chen, J., et al. (2021c) Contrasting short-term responses of soil heterotrophic and autotrophic respiration to biochar-based and chemical fertilizers in a subtropical Moso bamboo plantation. Applied Soil Ecology, 157, 103758. https://doi.org/10.1016/j.apsoil.2020.103758.

Zhang, S., Zhou, J., Chen, J., Ge, T., Cai, Y., Yu, B., et al. (2023) Changes in soil CO_2 and N_2O emissions in response to urea and biochar-based urea in a subtropical Moso bamboo forest. Soil and Tillage Research, 228, 105625. https://doi.org/10.1016/j.still.2022.105625.

Zhao, J. C., Su, W. H., Fan, S. H., Cai, C. J., Zhu, X. W., Peng, C., et al. (2016) Effects of various fertilization depths on ammonia volatilization in Moso bamboo (Phyllostachys edulis) forests. Plant, Soil and Environment, 62, 128–134. https://doi.org/10.17221/733/2015-PSE.

Zhao, J., Su, W., Fan, S., Cai, C., Su, H. and Zeng, X. (2019) Ammonia volatilization and nitrogen runoff losses from moso bamboo forests after different fertilization practices. Canadian Journal of Forest Research, 49, 213–220. https://doi.org/10.1139/cjfr-2018-0017.

Zhao, Z., Tao, C., Liu, X., Cheng, X., Zhou, C., Huang, S., et al. (2024) Effects of Different Management Measures on Carbon Stocks and Soil Carbon Stocks in Moso Bamboo Forests: Meta-Analysis and Control Experiment. Forests, 15, 496. https://doi.org/10.3390/f15030496.

Zheng, Y., Liu, X., Cai, Y., Shao, Q., Zhu, W. and Lin, X. (2022) Combined intensive management of fertilization, tillage, and organic material mulching regulate soil bacterial communities and functional capacities by altering soil potassium and pH in a Moso bamboo forest. Frontiers in Microbiology, 13, 944874. https://doi.org/10.3389/fmicb.2022.944874.

Zheng, X., Xu, A., Lin, Y., Wang, H., Ding, H., Wu, Y., et al. (2024) Long-term nitrogen deposition alters the soil bacterial community structure but has little effect on fungal communities. Global Ecology and Conservation, 54, e03080. https://doi.org/10.1016/j.gecco.2024.e03080.

Zhou, J., Qu, T., Li, Y., Van Zwieten, L., Wang, H., Chen, J., et al. (2021) Biochar-based fertilizer decreased while chemical fertilizer increased soil N_2O emissions in a subtropical Moso bamboo plantation. CATENA, 202, 105257. https://doi.org/10.1016/j.catena.2021.105257.

Zhou, J., Tang, C., Kuzyakov, Y., Vancov, T., Fang, Y., Song, X., et al. (2024) Biochar-based urea increases soil methane uptake in a subtropical forest. Geoderma, 449, 116994. https://doi.org/10.1016/j.geoderma.2024.116994.

Zhou, J., Tang, C., Vancov, T., Fu, S., Fang, Y., Ge, T., Dong, Y., Luo, Y., Yu, B., Cai, Y., White, J. C. and Li, Y. (2026) Biochar mitigates the suppressive effects of nitrogen deposition on soil methane uptake in a subtropical forest. Agriculture, Ecosystems & Environment, 395, 109951. https://doi.org/10.1016/j.agee.2025.109951.
